# Temporal Genetic Modifications after Controlled Cortical Impact—Understanding Traumatic Brain Injury through a Systematic Network Approach

**DOI:** 10.3390/ijms17020216

**Published:** 2016-02-06

**Authors:** Yung-Hao Wong, Chia-Chou Wu, John Chung-Che Wu, Hsien-Yong Lai, Kai-Yun Chen, Bo-Ren Jheng, Mien-Cheng Chen, Tzu-Hao Chang, Bor-Sen Chen

**Affiliations:** 1 College of Mechanical and Electronic Engineering, Fujian Agriculture and Forestry University, Fujian 350002, China; syslab.yvonwong@gmail.com; 2 Laboratory of Control and Systems Biology, Department of Electrical Engineering, National Tsing Hua University, Hsinchu 300, Taiwan; d9761820@oz.nthu.edu.tw (C.-C.W.); jbrpo810716@gmail.com (B.-R.J.); 3 Institute of Biomedical Science, National Chung Hsing University, Taichung 402, Taiwan; 4 Department of Neurosurgery, Taipei Medical University Hospital, Taipei Medical University, Taipei 110, Taiwan; dr.jcwu@gmail.com; 5 Institution Review Board (IRB), Christian Mennonite Hospital, Hualien 970, Taiwan; hsienyonglei@gmail.com; 6 Graduate Institute of Neural Regenerative Medicine, College of Medical Science and Technology, Taipei Medical University, Taipei 110, Taiwan; kychen08@tmu.edu.tw; 7 Division of Cardiology, Kaohsiung Chang Gung Memorial Hospital, College of Medicine, Chang Gung University, Kaohsiung City 833, Taiwan; chenmien@ms76.hinet.net; 8 Graduate Institute of Biomedical Informatics, Taipei Medical University, Taipei 110, Taiwan

**Keywords:** brain injury, network biomarker, systems biology, protein–protein interaction, drug target, cell cycle

## Abstract

Traumatic brain injury (TBI) is a primary injury caused by external physical force and also a secondary injury caused by biological processes such as metabolic, cellular, and other molecular events that eventually lead to brain cell death, tissue and nerve damage, and atrophy. It is a common disease process (as opposed to an event) that causes disabilities and high death rates. In order to treat all the repercussions of this injury, treatment becomes increasingly complex and difficult throughout the evolution of a TBI. Using high-throughput microarray data, we developed a systems biology approach to explore potential molecular mechanisms at four time points post-TBI (4, 8, 24, and 72 h), using a controlled cortical impact (CCI) model. We identified 27, 50, 48, and 59 significant proteins as network biomarkers at these four time points, respectively. We present their network structures to illustrate the protein–protein interactions (PPIs). We also identified UBC (Ubiquitin C), SUMO1, CDKN1A (cyclindependent kinase inhibitor 1A), and MYC as the core network biomarkers at the four time points, respectively. Using the functional analytical tool MetaCore™, we explored regulatory mechanisms and biological processes and conducted a statistical analysis of the four networks. The analytical results support some recent findings regarding TBI and provide additional guidance and directions for future research.

## 1. Introduction

Traumatic brain injury (TBI) is not only a primary injury, caused by external physical forces, but also a secondary injury caused by a series of biological processes. These include metabolic, cellular, and other molecular events that eventually lead to the death of brain cells, tissue and nerve damage, and atrophy [1]. Due to the importance of the brain, damage to this organ usually causes particularly complex symptoms relative to other injuries. For example, if the blood–brain barrier (BBB), a selectively permeable barrier to the transport of crucial elements for the brain, is damaged by an external force, several complications can occur due to disrupted homeostasis of the brain [2]. In order to take all aspects of the consequences of a TBI into account, and as knowledge on TBIs develops, treatment has become extremely complex and increasingly difficult [3]. Immediately after a TBI, primary injuries resulting from the initial trauma cause rupture of blood vessels, hemorrhaging, structural deformation of tissues, and massive death of neurons and other cells at injured sites [4]. Treatment of the primary injuries of a TBI is similar to those of other traumas. As a result of advances in the treatment of emergency traumas, secondary injuries have become the main cause of morbidity and mortality in TBI patients. Several studies have therefore concentrated on the treatment of secondary injuries resulting from TBIs in an effort to reduce the extent and effect of these injuries.

Secondary injuries are influenced by the vascular perturbations, cerebral metabolic dysfunction, and inadequate cerebral oxygenation related to primary injuries [5]. Vascular perturbations can affect the permeability of the BBB, pinocytotic activity of endothelial cells, and the redistribution of ions and neurotransmitters [6,7]. Increased permeability of the BBB leads to edema and subsequent brain swelling, and can increase the risk of brain infections [8]. Intracellular accumulation of potassium and calcium ions can affect the functioning of neurons in information transduction and of mitochondria in metabolism [9]. Elevated glutamate can be observed as early as 5 min after experimental trauma, which may cause excitotoxicity after a TBI [10] and can also affect mitochondrial functioning through oxidative stress [6]. Increased oxidative stress following a TBI is directly related to the pathogenesis of TBIs. Several oxidative markers, including reduced glutathione (GSH), the GSH/oxidized glutathione (GSSG) ratio, glutathione peroxidase (GPx), glutathione reductase (GR), glutathione-*S*-transferase (GST), glucose-6-phosphate dehydrogenase (G-6PD), superoxide dismutase (SOD), and catalase (CAT) are observed after a TBI. Hence, development of antioxidant strategies is of primary interest in ongoing efforts to optimize brain injury treatment [11]. Recently, inflammation was also recognized as a critical element in recovering from a TBI provided that TBI treatment is an option [12]. Inflammation is a general response to external insults and is also modulated by oxidative stress, mitochondrion-mediated metabolism, and cellular states [5]. An interwoven network of a wide variety of functions complicates TBIs and impedes the development of efficient treatments. Therefore, a systematic perspective of the evolution of a TBI is needed to provide a global view of molecular interactions.

In this study, we used a systems biology approach to elucidate protein–protein interactions (PPIs) very soon (4 h) and longer (8, 24, and 72 h) after a TBI [13]. A systems biology approach can provide a systematic view on the interwoven network of functions and interactions between the proteins involved in these complex networks, which are regarded as systems with sub-units (e.g., proteins) connected as a whole. Many PPIs can form PPI networks (PPINs), in which proteins are nodes and their interactions are the edges of the network. We also used a mathematical model with the microarray data to generate quantitative descriptions of the molecular interactions. By comparing injured and control networks, we were able to select some specific interactions and proteins as potential therapeutic or monitoring targets, according to their differential interactions. Connections between these targets and the physiological phenomena of a TBI provide insights into early interventions for TBIs.

## 2. Results and Discussion

We built the network biomarkers for the four time points post-TBI. We then used the commercial pathway analysis software MetaCore™ and the free network ontology analysis (NOA) web server to do a functional pathway analysis to reveal the underlying molecular mechanisms of TBI. The main results of this research are the network biomarkers generated by our model, *i.e.*, the proteins with the highest TBI relevance values (TRVs), and the network structure. However, MetaCore™ and NOA enabled us to add deeper medical and biology significance to our results and make them useful for identifying novel strategies for therapy or recovery processes. The powerful MetaCore™ software uses data mining and statistical methods to select valuable information from the biological and medical literature. It provides a more general perspective and enhanced interpretation of our research. The original results from our primary analysis thus form the core findings of this study, and the additional results generated by MetaCore™ and NOA can be viewed as supplementary information that is of wider benefit.

### 2.1. Evolution of Network Biomarkers at the Four Post-Traumatic Brain Injury (TBI) Time Points

**Figure 1:**
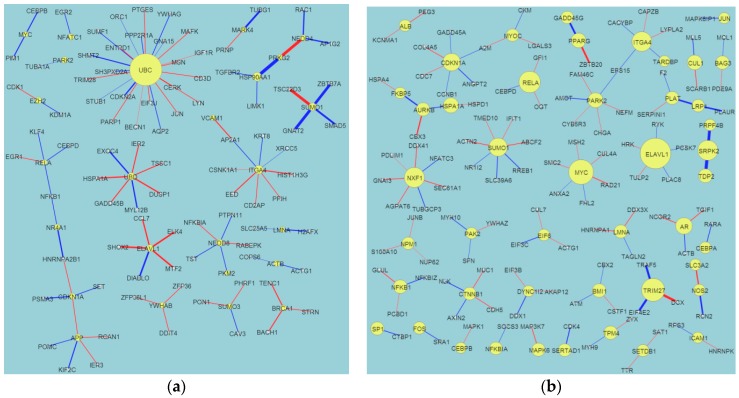

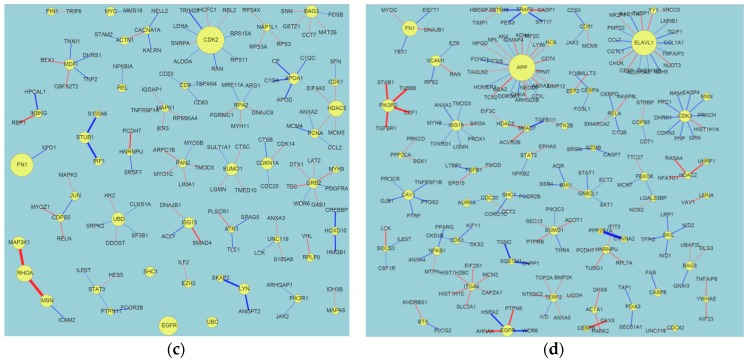
The constructed traumatic brain injury (TBI) differential protein–protein interaction networks (PPINs; DPPINs) for four time points post-TBI. These figures include edge and node information. The DPPIN is the difference between the TBI PPIN (TPPIN) and non-TBI PPIN (NPPIN). The size of nodes depends on the TBI relevance value (TRV). The proteins with the highest TRVs are defined as the network markers. These figures were created using Cytoscape. (**a**) 4 h; (**b**) 8 h; (**c**) 24 h; (**d**) 72 h. Blue edge means the difference in interaction activity ≤ mean − STD; Red edge means the difference in the interaction activity ≥ mean + STD; Yellow node means the protein with significant CRV.

We built a Differential PPIN (DPPIN) for each of the four post-TBI time points (4, 8, 24, and 72 h) (Figure 1) and calculated the TRV of each protein in the four networks (Table 1). The network diagrams (Figure 1) contain more information than just the TRVs, such as that encoded by the edges and nodes. Node size represents the TRV of each protein, and edge width the interaction ability between the two proteins. Red and blue edges respectively indicate positive and negative values of *d_ij_* in Equation (7). We identified statistically significant network marker proteins for the four post-TBI stages by screening the TRV *p*-values. As in our previous study on stroke, we wanted to reveal the repair mechanisms that operate at these four post-TBI time points. After fold change screening (FC (Fold Change) > 1.5), we identified 27, 50, 48, and 59 significant proteins at 4, 8, 24, and 72 h post-TBI, respectively. We only list the top 20 for each time point in Table 1, but provide the full lists in a supplementary table. Their corresponding TRVs were in the ranges 4.5–64.5, 4.9–17, 5.3–30.4, and 5.1–34.2, respectively. We used these significant proteins and their PPIs to construct network markers for the four post-TBI time points. The protein relevance values for TBI were much smaller than those we calculated for carcinogenesis in our previous study on cancer [9,10], and the cancer networks were much more complex than the TBI networks. However, the TBI values and network structures were similar to those we identified for stroke. We do not discuss the UBC (Ubiquitin C), protein in this paper because it is a complex issue. *UBC* is a housekeeping gene for many different diseases, such as cancers, stroke, and TBI. Recent findings also demonstrate that Ubiquitin Carboxyl-Terminal Esterase L1 (Ubiquitin Thiolesterase, UCHL1) is a promising biomarker that physically interacts with UBC [14,15]. Our demonstration of UBC’s role in the first 72 h after TBI supports the significance of UCHL1 in TBI.

**Table 1:** The 20 proteins with the highest TBI relevance values (TRVs) at four time points post-TBI (traumatic brain injury). AvgExp: average expression; Log_2_ FC: log_2_ fold change. This table was generated using the Matlab program developed by our team according to the algorithms described in Section 3.3 and Supplementary Materials.

Protein	TRV	*p*-Value	TBI_AvgExp	Normal_AvgExp	Log_2_ FC
**4 h**					
UBC	64.77	<10^−9^	9545	20,349	−1.09
HSP90AA1	12.45	0.000167	11,942	22,341	−0.9
ITGA4	11.92	0.000167	172	280	−0.7
SUMO1	10.22	0.000167	8999	9258	−0.04
UBD	9.73	0.000333	126	209	−0.73
ELAVL1	8.33	0.000667	1549	1785	−0.2
APP	8.29	0.000667	9608	19,299	−1.01
NEDD8	8.1	0.000667	4903	5672	−0.21
PARK2	7.45	0.001833	47	167	−1.83
EZH2	7.42	0.002	633	362	0.8
CDKN1A	6.63	0.0045	3046	1920	0.67
ACTB	6.53	0.005	15,663	26,523	−0.76
NFATC1	6.53	0.005167	273	77	1.84
NEDD4	6.22	0.006833	4030	4698	−0.22
YWHAB	5.81	0.011333	2014	3474	−0.79
SUMO3	5.77	0.012333	16,117	13,002	0.31
NR4A1	5.75	0.0125	8539	3969	1.11
BRCA1	5.46	0.016667	82	216	−1.4
PRKG2	5.43	0.017667	29	60	−1.06
LMNA	5.25	0.021333	10,185	5878	0.79
**8 h**					
ELAVL1	16.97	0.000868	1609	1082	0.57
MYC	13.35	0.001157	356	96	1.9
TRIM27	12.28	0.00135	3114	2773	0.17
RELA	11.34	0.001446	6282	3408	0.88
MDM2	10.9	0.001543	2615	2317	0.17
NXF1	10.48	0.001543	1086	1240	−0.19
SRPK2	10.4	0.001639	1183	1232	−0.06
ITGA4	10.26	0.001639	138	231	−0.74
SUMO1	9.35	0.002314	9180	10,837	−0.24
CDKN1A	8.92	0.002893	3417	661	2.37
NEDD8	8.43	0.003857	4659	5525	−0.25
AR	8.35	0.003857	824	1585	−0.94
HSPA1A	7.5	0.005593	5663	2112	1.42
PARK2	7.34	0.006268	28	51	−0.87
CUL1	7.22	0.006847	4406	4936	−0.16
PPARG	7.17	0.007329	169	126	0.42
UBC	6.93	0.0081	26,887	29,005	−0.11
TDP2	6.69	0.00974	719	1230	−0.78
BMI1	6.58	0.010511	2465	3576	−0.54
NFKB1	6.49	0.011765	1896	1398	0.44
**24 h**					
CDK2	30.4	0.000563	612	302	1.02
FN1	25.07	0.000805	2652	1701	0.64
EGFR	20.02	0.001689	315	197	0.68
RHOA	17.25	0.002816	4082	4140	−0.02
HDAC5	16.31	0.003057	4914	7484	−0.61
UBD	13.24	0.004023	97	219	−1.17
MAP3K1	12.48	0.004586	876	364	1.27
MSN	12.27	0.004666	4631	1331	1.8
BAG3	12.13	0.004747	1206	419	1.52
SUMO1	12	0.004747	9074	10,206	−0.17
CDKN1A	11.59	0.004988	4037	740	2.45
STUB1	10.71	0.005632	6156	5743	0.1
LYN	10.59	0.006034	350	312	0.17
APOA1	10.52	0.006195	102	63	0.71
UBC	10.27	0.006436	30,215	27,824	0.12
ISG15	10.26	0.006436	1137	469	1.28
MDFI	9.46	0.007723	659	547	0.27
SHC1	9.31	0.008045	788	441	0.84
GRB2	8.91	0.008286	3458	3862	−0.16
MYC	8.41	0.00901	251	120	1.06
**72 h**					
APP	34.02	<10^−9^	31,743	38,874	−0.29
ELAVL1	31.92	<10^−9^	1937	1169	0.73
FN1	18.2	0.000309	3441	1562	1.14
PIK3R2	14.17	0.000386	1495	1625	−0.12
CDK2	13.29	0.000464	529	383	0.47
EGFR	12.62	0.000464	325	384	−0.24
VCAM1	12.3	0.000541	1691	994	0.77
ISG15	11.52	0.000696	1651	628	1.4
UBC	11.33	0.00085	31,436	28,733	0.13
CAV1	10.69	0.001005	2024	802	1.34
TRAF2	9.53	0.001468	1479	1636	−0.15
BAG3	8.94	0.002473	1205	506	1.25
SUMO1	8.94	0.002473	7368	9430	−0.36
NFKB1	8.7	0.002782	2636	1476	0.84
FBXO6	8.6	0.003014	5920	4444	0.41
TERF2	8.52	0.003091	878	1464	−0.74
HNRNPU	8.43	0.003246	150	291	−0.95
GNB2L1	8.26	0.003632	17,568	10,465	0.75
SKIL	7.99	0.004405	149	43	1.79
AURKA	7.89	0.004637	518	175	1.56

We also identified four significant proteins of core network biomarkers (intersections) for the four post-TBI time points: UBC, SUMO1, CDKN1A, and MYC (Table 2). Core and specific network biomarkers are important when discussing evolutionary processes. The discovery of these significant proteins, which are consistent throughout the four post-TBI time points, is especially interesting as Natale *et al.* [16] show MYC was the only one of these proteins identified as common to the different TBI models using controlled cortical impact (CCI) and fluid percussion injury (FPI). To comprehensively discuss the evolutionary behavior of TBI, we also summarized the core network (intersections) of each adjacent time point, *i.e.*, 4–8, 8–24, and 24–72 h.

**Table 2:** The four significant proteins of core network biomarkers (intersections) for the four post-TBI time points. AvgExp: average expression; Log_2_ FC: log_2_ fold change; Time point: number of hours post-TBI. This table was generated by the Matlab program developed by our team according to the algorithms described in Section 3.3 and Supplementary Materials.

Gene	Time Point (h)	CRV_Value	*p*-Value	Case_AvgExp	Control_AvgExp	Log_2_ FC
*UBC*	4	64.77	<10^−9^	9545	20,349	−1.09
*UBC*	8	6.93	0.0081	26,887	29,005	−0.11
*UBC*	24	10.27	0.006436	30,215	27,824	0.12
*UBC*	72	11.33	0.00085	31,436	28,733	0.13
*SUMO1*	4	10.22	0.000167	8999	9258	−0.04
*SUMO1*	8	9.35	0.002314	9180	10,837	−0.24
*SUMO1*	24	12	0.004747	9074	10,206	−0.17
*SUMO1*	72	8.94	0.002473	7368	9430	−0.36
*CDKN1A*	4	6.63	0.0045	3046	1920	0.67
*CDKN1A*	8	8.92	0.002893	3417	661	2.37
*CDKN1A*	24	11.59	0.004988	4037	740	2.45
*CDKN1A*	72	6.68	0.010974	2257	757	1.58
*MYC*	4	4.84	0.035667	281	144	0.96
*MYC*	8	13.35	0.001157	356	96	1.9
*MYC*	24	8.41	0.00901	251	120	1.06
*MYC*	72	5.93	0.022411	268	63	2.08

Of the biomarkers identified in our analysis, UBC has also been identified in other systems biology reviews of TBI datasets. As Feala [17] mentions, “Animal experiments do not always reproduce the same results across studies. This is primarily because of variations in animal species, injury type and severity, time course of collection, and sampled tissue”. For example, MAPT (Microtubule Associated Protein Tau), also known as Tau (Tau proteins are proteins that stabilize microtubules), appears to be a promising biomarker, yet it does not appear to increase in mTBI (mild traumatic brain injury) scenarios. The change in genetic profiles across different time points was especially problematic for us because a practical biomarker should ideally be present and examinable at different time points after the injury. Of the four proteins common among the different time points, only UBC was mentioned in Feala’s article [17]. We feel that other proteins that are persistent throughout the course of sampling should also receive additional attention.

The unique genetic profile for UBC, SUMO1, and CDKN1A at the four time points for CCI suggests the need to further assess current biological modeling designs. We have previously found that even the sham control groups require more precise treatment in CCI experimentation [17] and have proposed that unicortical drilling should be performed on the control group. Since improved biological modeling requires additional funding and repeating previous experiments, we have used a systems biology approach to re-analyze the controversial penumbra region. Given that the penumbra region contains salvageable brain tissue and that it lies between the heavily-injured region and the nearly-normal region, we feel that appropriate analysis of this region should disclose valuable information and targets for treatment.

Our analyses revealed significant information regarding the recovery and damage processes in the post-TBI stages. This information provides clues for selecting novel drug targets for therapeutic and recovery processes. Because of article length restrictions, we present these results in a supplementary table.

### 2.2. Network Structure Interpretation of the Four Post-TBI Time Points

Figure 1 and Table 1 show the difference between the network structures of the four time points. At 4 h post-TBI, the UBC node dominated the network (TRV = 64.8), while the TRVs of the other nodes were all <15. We discuss the next two most important proteins, heat shock protein HSP90AA1 (Heat shock protein 90) and ITGA4 (integrin α-4 precursor gene) (TRV = 12.4 and 11.9, respectively), below. At 8 h, there were no TRVs greater than 20. However, ELAVL1 (embryonic lethal abnormal vision-like protein 1) (TRV = 17.0) appeared to be a significant protein of neuron-related diseases. At 24 h, CDK2 (cyclin-dependent kinase-2), FN1, and epithelial growth factor receptor (EGFR) (TRV = 30.4, 25, and 20, respectively) dominated the network. We discuss these significant nodes below. Finally, at 72 h, APP (amyloid precursors protein) and ELAVL1 (TRV = 34 and 31.9, respectively) dominated the network. The color and width of the edges in Figure 1 reveal some information about the regulatory mechanisms of the proteins, but the model we used in this research focused on PPIs, not genetic regulatory networks (GRNs). We think that the significant nodes we identified are more like “hubs” than drivers. Our team has also developed GRN models [18], but due to limited data types it was not appropriate to apply a GRN model to this dataset.

We discuss the five aforementioned proteins here.

(i) HSP90AA1: White *et al.* [19] discussed the relationship between gene expression patterns post-TBI and the inflammatory response. They identified the following significant genes: HSP90AA1, ERAP1 (endoplasmic reticulum aminopeptidase 1), PSMB9 (proteasome subunit beta type-9), CBL (calcineurin B-like), BTK (Bruton’s tyrosine kinase), RORA (retinoic acid receptor-related orphan receptor alpha), THRA (thyroid hormone receptor alpha), and ITGA5 (Integrin, alpha 5, fibronectin receptor).
(ii) *ITGA4* (Integrin, α-4 precursor gene): White *et al.* identified ITGA5, but not ITGA4 [19]. They are in the same family. ITGA4 is always related to inflammation caused by stroke or TBI. Fulmer discussed epilepsy drug effects on these proteins [20].
(iii) CDK2: Zhang discussed the relationships between the expression of BAD, CDK2, and STAT3, and brain function after a TBI in rats [21].
(iv) FN1: White *et al.* discussed the complex behaviors of FN1, such as its extracellular matrix/cell adhesion (FN1, matrix metalloproteinases (MMPs), and ICAM1) module, its fold changes in a gene interaction hierarchy (GIH) analysis, and others [19].
(v) EGFR: This is a well-known cancer oncogene and the vascular EGFR (VEGFR) receptor is always reported in brain injuries [22]. It is a novel clue for us to identify the relationship between EGFR and TBI.

### 2.3. Comparison with Our Previous Results for Stroke

We compare our results with our recently conducted work on human stroke [23]. We identified five significant proteins common to both stroke and TBI: UBC, APP, NEDD8, PAN2, and EVAVL1.

APP activates voltage-dependent calcium channels and may induce neuronal apoptosis. Its protein product binds growth factor receptor and plays a role in the regulation of peptidase activator and acetylcholine receptor activities. It is involved in the positive regulation of peptidase activity, locomotor behavior, and axon cargo transport. It also participates in the glypican signaling pathway and Alzheimer disease (AD) pathway [24,25]. It is involved in many cellular behaviors and thus forms one of the key hubs in TBI.

NEDD8 (neural precursor cell expressed) encodes a protein that exhibits ubiquitin protein ligase binding. It is involved in protein neddylation. It participates in the p53 signaling pathway, the neddylation pathway, and the cullin-dependent proteasome degradation pathway. It is also associated with Parkinson’s disease [25,26].

There are few reports that PAN2 is directly related to TBI or stroke, so this could be a novel target for therapy. The ELAVL1/Hu family of RBPs (RIM binding proteins) plays a key role in neuroscience. Skliris *et al.* [27] discuss how neuroprotective behavior requires the functions of the RNA-binding protein, HuR, and give a full description of the mechanisms of ELAVL1/HuR. Usually, three members of this family (HuB/HEL-N1/ELAVL2, HuC/ELAVL3, and HuD/ELAVL4) are expressed by neurons, but ELAVL1/HuR is always expressed in both neuronal and non-neuronal tissues. These findings concur with descriptions of the involvement of synaptic dysfunction and cytoarchitectural degradation in stroke [28].

### 2.4. MetaCore™ Results

We used the MetaCore™ Analyze Networks (AN) algorithm to analyze the network biomarkers that were unique to or common to all or some of the networks we identified for the four time points post-TBI (Table 3). The detailed algorithm is given in Supplementary Material S0, showing that completely different cellular behaviors are operating at the four time points. Briefly, most biomarkers at 4 h post-TBI were related to cell activation and signaling behaviors. In contrast, most at 8 h post-TBI were related to immune response. At 24 h post-TBI, most were related to DNA behavior, whereas in the final 72 h post-TBI stage, most were related to regulation and apoptotic behaviors. The evolution of cellular behavior at these four time points post-TBI is thus clear.

A canonical pathway in MetaCore™ represents a set of consecutive signals, or metabolic transformations, confirmed as a whole by experimental data or by inferred relationships. They are linear sets of carefully defined steps that form a map; in MetaCore™, complete biochemical pathways or signaling cascades in the commonly accepted sense correspond to maps. Over 70,000 pathways are stored in MetaBase™, which are mainly used for network generation and visualized as canonical pathway maps. The AN algorithm creates a large network and divides it into smaller sub-networks that can be separately built. Networks are more interactive than pathway maps and can sometimes be more complex.

**Table 3:** Analysis of networks using MetaCore™. The gene content of the uploaded files was used as the input list for generating biological networks using the Analyze Networks (AN) algorithm with default settings. This is a variant of the shortest paths algorithm, with the following main parameters: (1) relative enrichment with the uploaded data and (2) relative saturation of networks with canonical pathways. These networks are built on the fly and are unique to the uploaded data. In this workflow, the networks are prioritized based on the number of fragments of canonical pathways in the network. S: size; T: target; P: pathways; G: gScore.

No.	Processes	S	T	P	G
**Common to All Four Networks**					
1	Viral transcription (97.1%), viral genome expression (97.1%), translational termination (97.1%), cellular protein complex disassembly (97.1%), SRP (signal recognition particle)-dependent cotranslational protein targeting to membrane (97.1%).	38	1	0	11.39
**Common to Two or Three of the Networks**					
1	Positive regulation of nucleobase-containing compound metabolic process (75.5%), positive regulation of biosynthetic process (77.6%), enzyme-linked receptor protein signaling pathway (65.3%), positive regulation of nitrogen compound metabolic process (75.5%), positive regulation of macromolecule biosynthetic process (73.5%).	50	16	0	69.15
2	Positive regulation of response to stimulus (60.4%), regulation of response to stimulus (75.0%), response to organic substance (72.9%), transmembrane receptor protein tyrosine kinase signaling pathway (45.8%), response to hormone stimulus (56.2%).	50	14	0	56.71
3	Cell surface receptor signaling pathway (96.0%), signal transduction (96.0%), signaling (96.0%), single organism signaling (96.0%), cell communication (96.0%).	50	2	30	45.94
**Unique to the 4 h Post-TBI Network**					
1	Cell activation (54.0%), signal transduction (94.0%), signaling (96.0%), single organism signaling (96.0%), response to wounding (62.0%).	50	6	0	43.25
2	G-protein coupled receptor signaling pathway (71.4%), neuropeptide signaling pathway (30.6%), G-protein coupled receptor signaling pathway, coupled to cyclic nucleotide second messenger (32.7%), cell surface receptor signaling pathway (75.5%), chemokine-mediated signaling pathway (20.4%).	50	5	0	36.02
3	Axis specification (38.0%), canonical Wnt receptor signaling pathway (36.0%), pattern specification process (46.0%), Wnt receptor signaling pathway (38.0%), anterior/posterior pattern specification (36.0%).	50	4	0	28.78
**Unique to the 8 h Post-TBI Network**					
1	Response to abiotic stimulus (61.7%), regulation of apoptotic process (63.8%), regulation of programmed cell death (63.8%), positive regulation of cellular process (85.1%), positive regulation of metabolic process (74.5%).	50	9	0	41.50
2	Positive regulation of immune response (43.8%), positive regulation of response to stimulus (58.3%), regulation of response to stress (52.1%), regulation of immune response (47.9%), TRIF (Toll/IL-1 receptor domain-containing adapter inducing interferon-β)-dependent toll-like receptor signaling pathway (27.1%).	50	8	0	36.87
3	Immune response-activating signal transduction (25.0%), immune response-regulating signaling pathway (25.0%), T cell costimulation (17.5%), lymphocyte costimulation (17.5%), cellular defense response (17.5%).	50	5	2	25.40
**Unique to the 24 h Post-TBI Network**					
1	Double-strand break repair via synthesis-dependent strand annealing (100.0%), DNA recombinase assembly (100.0%), DNA excision (100.0%), telomere maintenance via semi-conservative replication (100.0%), nucleotide-excision repair, DNA gap filling (100.0%).	4	2	0	33.30
2	Lipoprotein metabolic process (46.2%), protein-lipid complex assembly (34.6%), plasma lipoprotein particle assembly (34.6%), lipid transport (50.0%), lipid localization (50.0%).	49	6	0	30.34
3	Immune response (56.5%), immune system process (67.4%), positive regulation of response to stimulus (58.7%), activation of immune response (39.1%), positive regulation of immune system process (47.8%).	50	6	0	28.11
**Unique to the 72 h Post-TBI Network**					
1	Positive regulation of biological process (98.0%), positive regulation of cellular process (90.0%), enzyme linked receptor protein signaling pathway (56.0%), regulation of immune system process (60.0%), regulation of response to stimulus (76.0%).	50	13	0	65.57
2	Positive regulation of apoptotic process (58.0%), positive regulation of programmed cell death (58.0%), positive regulation of cell death (58.0%), positive regulation of biological process (90.0%), regulation of apoptotic process (66.0%).	50	11	0	55.45
3	Enzyme linked receptor protein signaling pathway (63.8%), membrane protein intracellular domain proteolysis (25.5%), nerve growth factor receptor signaling pathway (40.4%), membrane protein proteolysis (25.5%), transmembrane receptor protein tyrosine kinase signaling pathway (46.8%).	50	10	0	50.91

#### 2.4.1. Statistical Interpretation of MetaCore™

**Figure 2:**
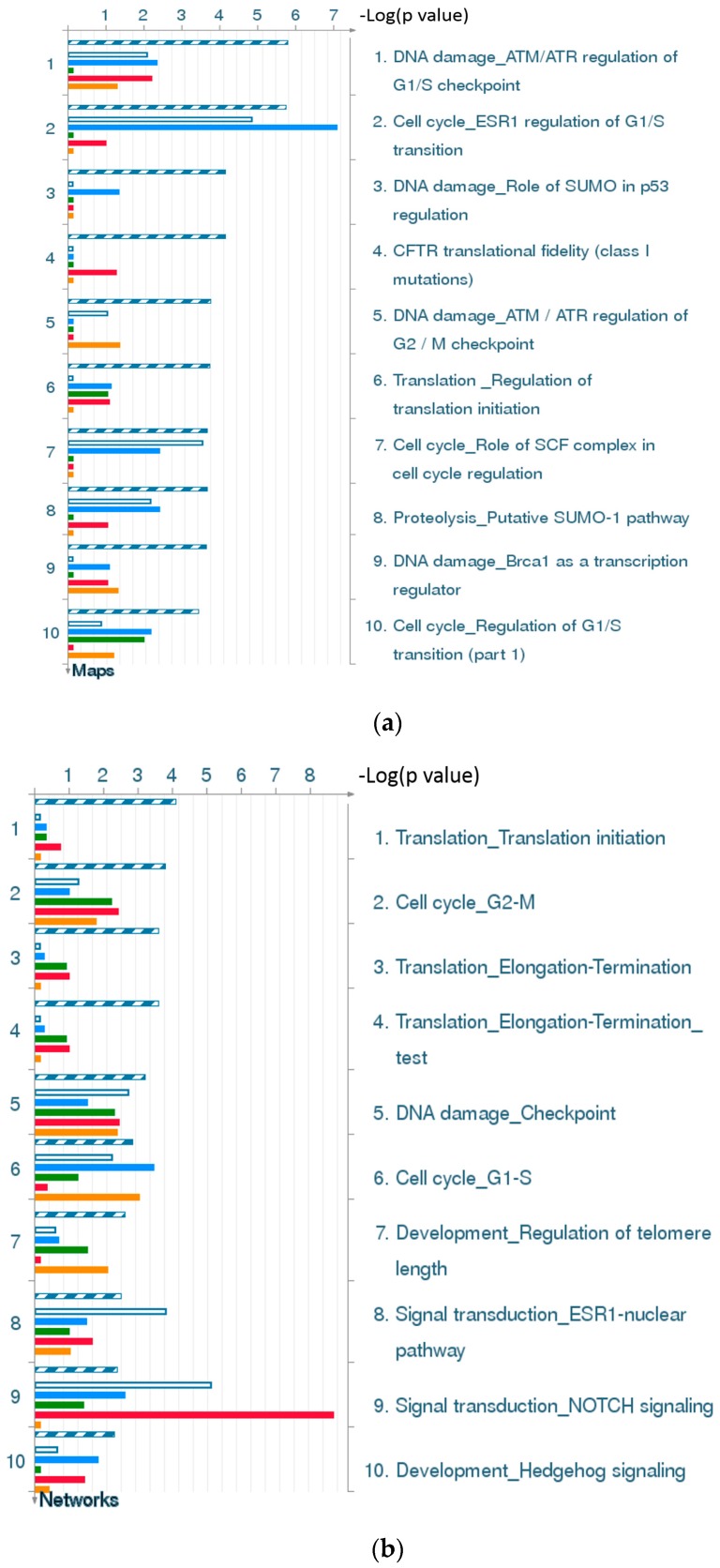

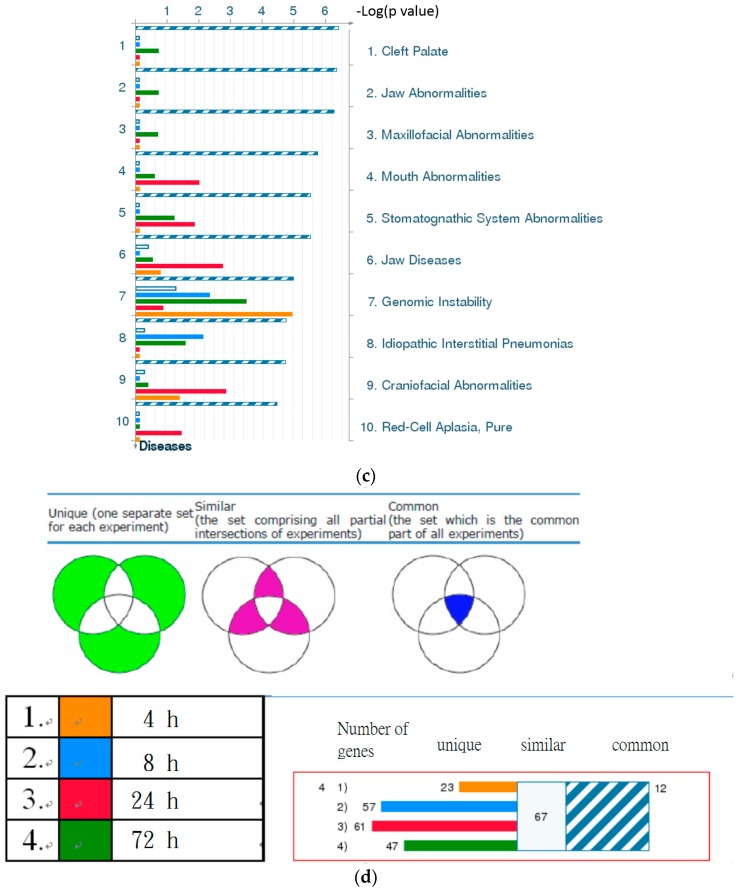
Results of the MetaCore™ analysis for all four time points post-TBI (4 h, 8 h, 24 h, and 72 h). All results are sorted according to those most strongly represented in the “common” set. (**a**) Pathway maps. Canonical pathway maps represent a set of signaling and metabolic maps that comprehensively cover human biological functioning. All maps are created by Thomson Reuters scientists using a high quality manual curation process based on published peer reviewed literature. Experimental data are visualized on the maps as blue (for downregulation) and red (upregulation) histograms. The height of the histogram corresponds to the relative expression value for a particular gene/protein; (**b**) Process networks. The content of these cellular and molecular processes is defined and annotated by Thomson Reuters scientists. Each process represents a pre-set network of protein interactions characteristic of that process; (**c**) Related diseases (determined based on biomarkers). Related disease folders are organized into a hierarchical tree. Gene content may vary greatly between such complex diseases as cancers and some Mendelian diseases. Also, coverage of different diseases in literature is skewed. These two factors may affect *p-*value prioritization for diseases; (**d**) Figure legend giving the meanings of the colors and statistical interpretation of the above three figures. These figures were generated by MetaCore™.

Figure 2d and Figure 4d are statistical interpretation of MetaCore™. The comparison tool, after the user has set the various options, generates a number of intersecting sets. The number of sets generated depends on the number of active experiments (in this case, time points post-TBI under consideration, *i.e.*, four). They overlap where they have network objects in common. The different elements of the resulting Venn diagram comprise of the following: (1) a “common” area in the middle, including the network objects found in all the experiments (in this case, at all four time points); (2) an area “unique” to each experiment, consisting of objects unique to that experiment; and (3) one or more sets of objects common to two or more experiments, but not all (MetaCore™).

#### 2.4.2. Three Statistical Results for the Four Time Points Post-TBI

Figure 2a gives the top 10 pathway maps. Figure 2b gives the top 10 process networks. They are highly related to the cell cycle, DNA damage, and signal transduction. These pathways and process networks may provide clues for new uses of conventional drugs. From the literature review, we also found that it was important to pay attention to the relationship between the cell cycle and TBI [29]. Simone *et al.* discuss the fact that cell cycle inhibition provides neuroprotection and reduces glial proliferation and scar formation after traumatic brain injury. This article has been cited more than 250 times, and therefore the discussion of the relationship of the cell cycle and TBI is very important. Figure 2c gives results for related diseases. While one can make comparisons of common hidden embryological and molecular mechanisms of these diseases, the correlation and embryological relationship amongst TBI and these diseases, however, would require additional investigation and validation.

#### 2.4.3. The Highest-Scoring Pathway Map of Post-TBI-Related Biomarker Genes at the Four Time Points

**Figure 3:**
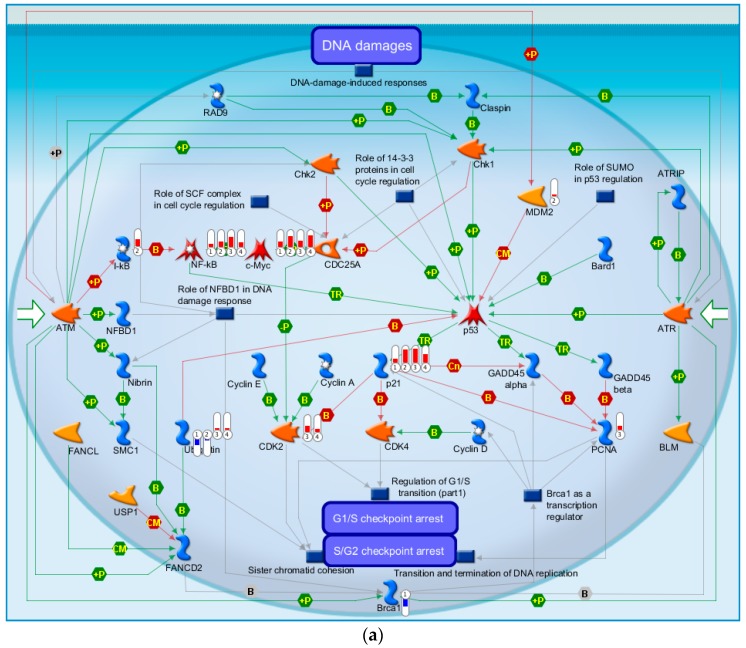

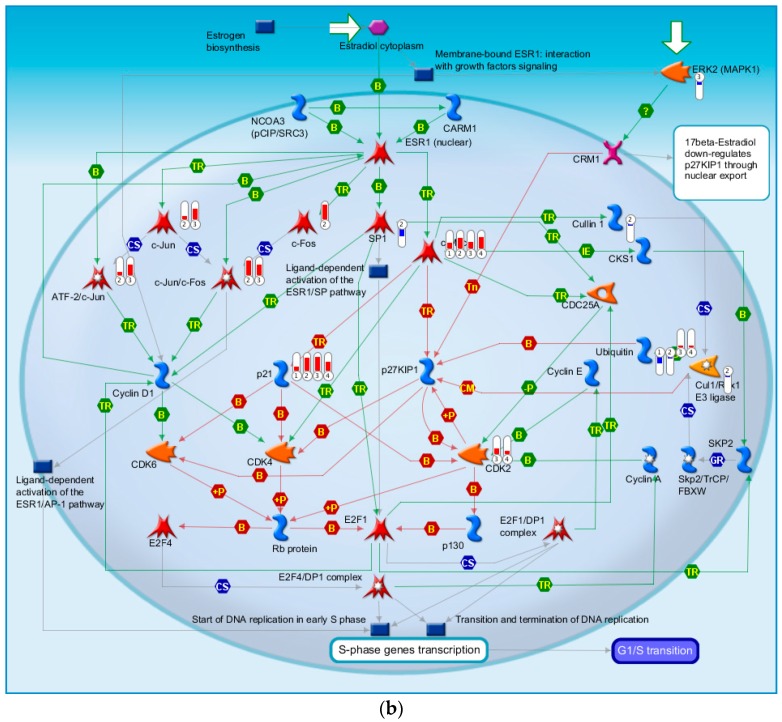
The two highest-scoring maps for all four time points post-TBI. (**a**) DNA damage ATM/ATR regulation of G1/S checkpoint; (**b**) Cell cycle ESR1 regulation of G1/S transition. These figures were generated by MetaCore™.

The highest-scoring pathway map we identified using MetaCore for the four time points was DNA damage ATM (Ataxia-telangiectasia mutated )/ATR (Ataxia-telangiectasia and Rad3-related) regulation of G1/S checkpoint (Figure 3a). The ATM-Chk2 (ataxia telangiectasia mutated–checkpoint kinase 2) and ATR-Chk1 pathways are two distinct kinase signaling cascades that primarily coordinate cellular responses to DNA damage. The DNA damage checkpoints may arrest or delay progression of cell cycles in response to DNA damage. Notably, NF-κB, c-MYC, and P21 were up-regulated at all four time points relative to the time at which the injury occurred. Ubiquitin was down-regulated at both 4 and 8 h and up-regulated at 24 and 72 h. BRCA1 was down-regulated and MDM2 and I-κB were up-regulated at 4 h. PCNA was up-regulated at 24 h and CDK2 was up-regulated at 24 and 72 h. The second highest-scoring map was Cell Cycle ESR1 regulation of G1/S transition, which is also a cell cycle associated pathway (Figure 3b). C-MYC and P21 were up-regulated at all four time points. ATF-2/c-Jun, c-Jun, and c-Jun/c-Fos were up-regulated at both 8 and 24 h. SP1, Cullin 1, and Cul1/Rbx1 E3 ligase were down-regulated at 8 h. CDK2 was up-regulated at both 24 and 72 h. These results show that our identified biomarker genes are strongly associated with the cell cycle and may play important roles in post-TBI regulation.

#### 2.4.4. Three Statistical Results for the Last Two Time Points Post-TBI

**Figure 4:**
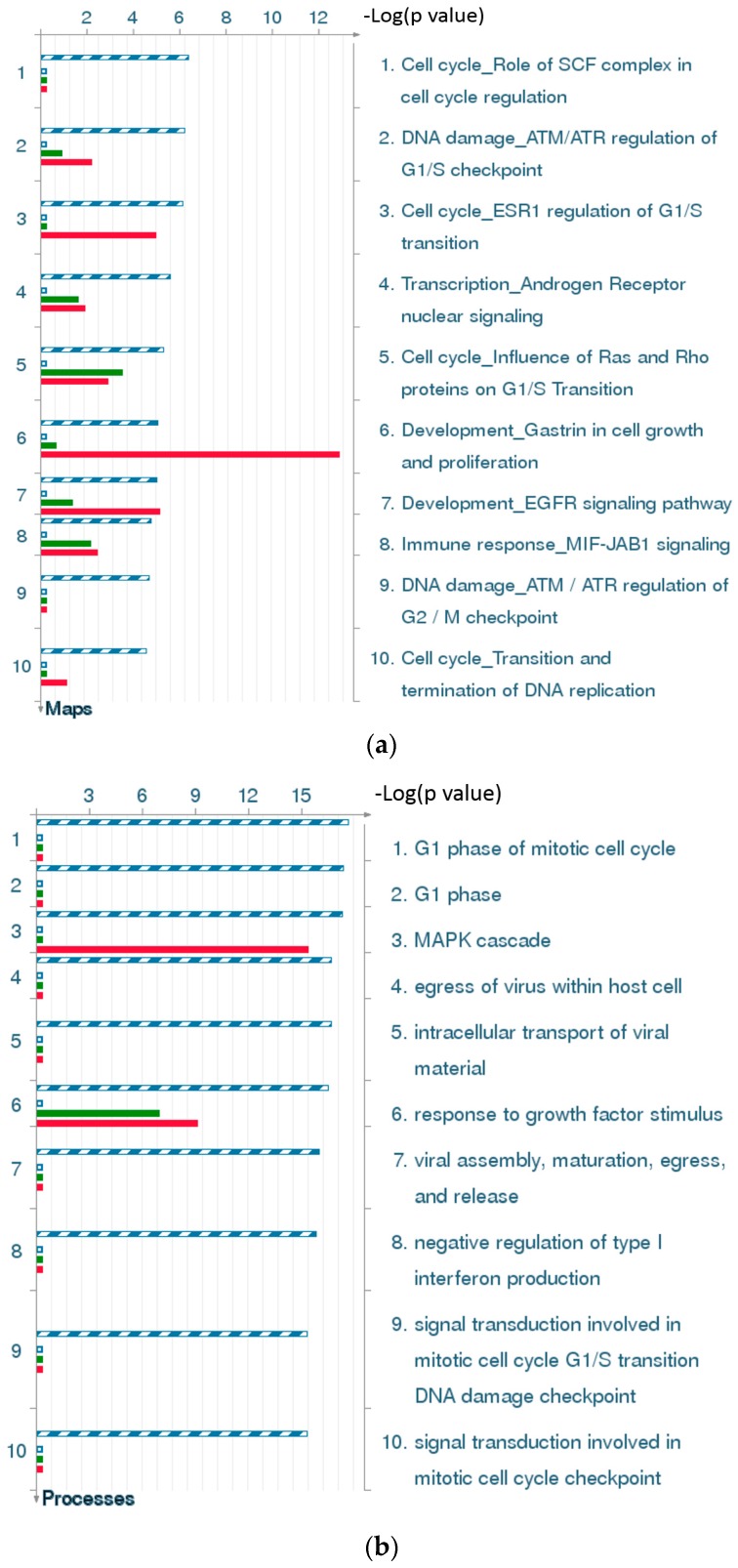

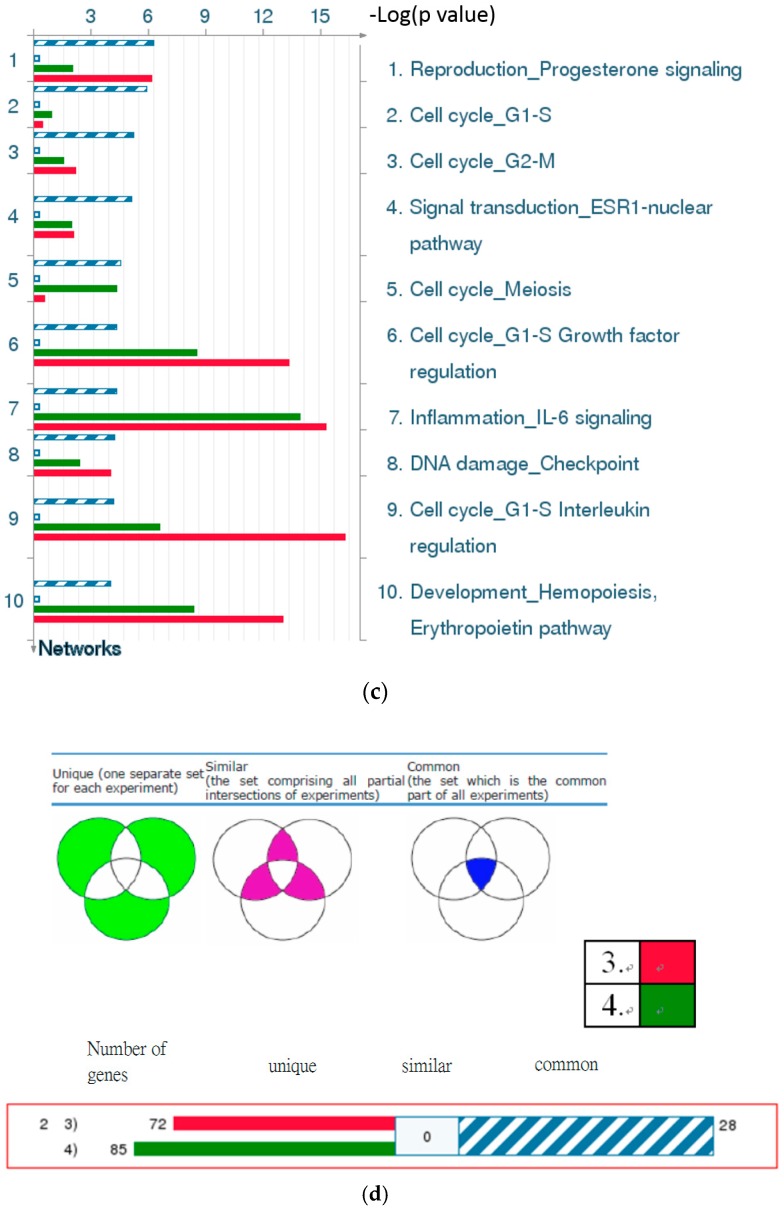
Results of the MetaCore™ analysis for the last two time points post-TBI (24 and 72 h). Results are sorted according to those most strongly represented in the “common” set. (**a**) Pathway maps. Canonical pathway maps represent a set of signaling and metabolic maps that comprehensively cover human biological functioning. All maps are created by Thomson Reuters scientists using a high quality manual curation process based on published peer reviewed literature. Experimental data is visualized on the maps as blue (for down-regulation) and red (up-regulation) histograms. The height of the histogram corresponds to the relative expression value for a particular gene/protein; (**b**) Gene ontology (GO) cellular processes. Since most GO processes have no gene/protein content, the “empty terms” are excluded from *p-*value calculations; (**c**) Process networks. The content of these cellular and molecular processes is defined and annotated by Thomson Reuters scientists. Each process represents a pre-set network of protein interactions characteristic for the process; (**d**) Figure legend giving the meanings of the colors and statistical interpretation of the above three figures. These figures were generated by MetaCore™.

The 10 top-scoring pathway maps (Figure 4a) and process networks (Figure 4c) for the last two time points post-TBI (24 and 72 h) are strongly related to the cell cycle, DNA damage, and signal transduction. The results regarding related diseases (Figure 4c) provide clues about new uses for conventional drugs and facilitate comparisons of the hidden molecular mechanisms common to some of these diseases.

#### 2.4.5. The Highest-Scoring Pathway Map of Post-TBI-Related Biomarker Genes at 24 and 72 h

**Figure 5:**
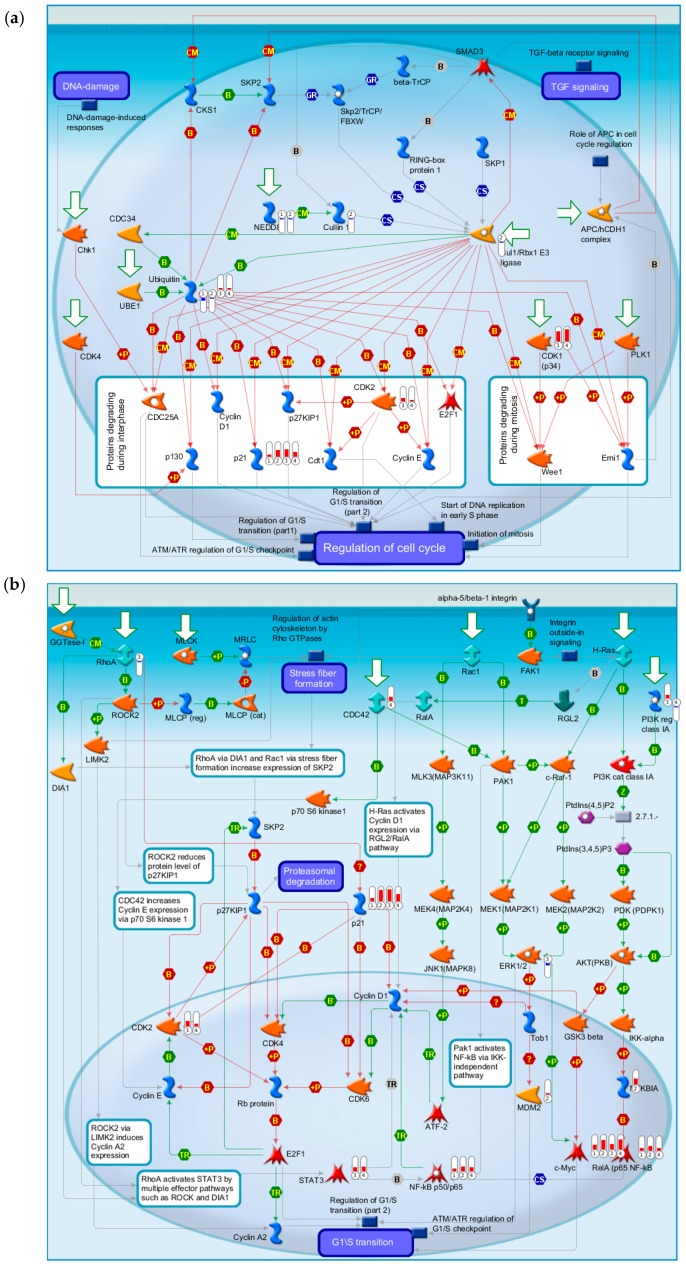
The highest-scoring and fifth highest-scoring pathway maps for the last two time points post-TBI (24 and 72 h). (**a**) Cell cycle: role of SCF complex in cell cycle regulation; (**b**) Cell cycle: Influence of Ras and Rho proteins on G1/S Transition. These figures were generated by MetaCore™.

The highest-scoring pathway map at 24 and 72 h, according to the MetaCore™ analysis, is Cell Cycle Role of SCF Complex (Skp, Cullin, F-box containing complex) in Cell Cycle Regulation (Figure 5a). The S-phase kinase-associated protein (SKP1)/Cullin/F-box (SCF) complex is one of the E3-ubiquitin ligases, and plays a critical role in the cell cycle. Surprisingly, ubiquitin was down-regulated at 4 and 8 h, but up-regulated at 24 and 72 h. CDK1 and CDK2 were up-regulated at 24 and 72 h, and P21 was up-regulated at all four time points. Another pathway related to the cell cycle, influence of Ras and Rho proteins on G1/S transition, was also identified as strongly associated with biomarker genes at 24 and 72 h (Figure 5b). These genes, such as *P21*, *CDK2*, *ERK1*/2, *MDM2*, *STAT3*, *RhoA*, *NF*-*κB*, might provide clues for deciphering the regulatory mechanisms of post-TBI processes at different time points.

#### 2.4.6. Top-Scoring AN (Analyze Networks Algorithm) Results for the Four Time Points Post-TBI

**Figure 6:**
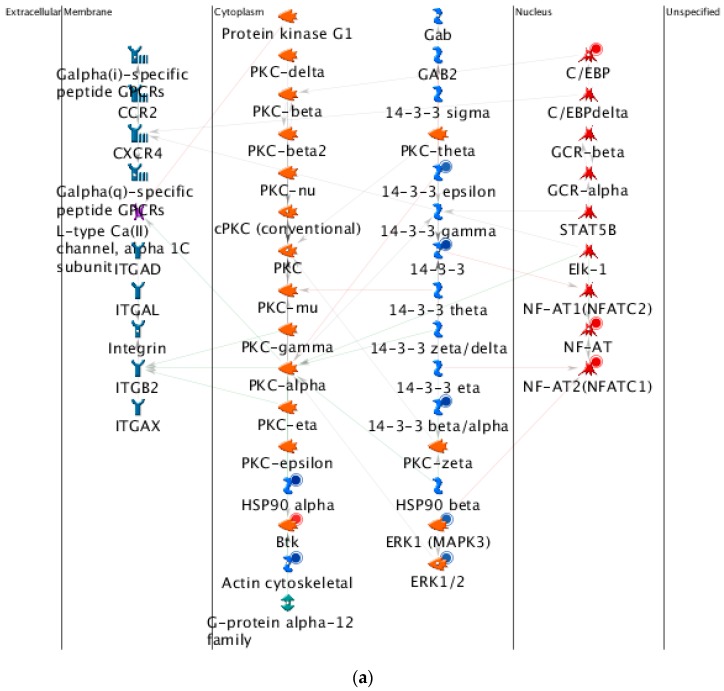

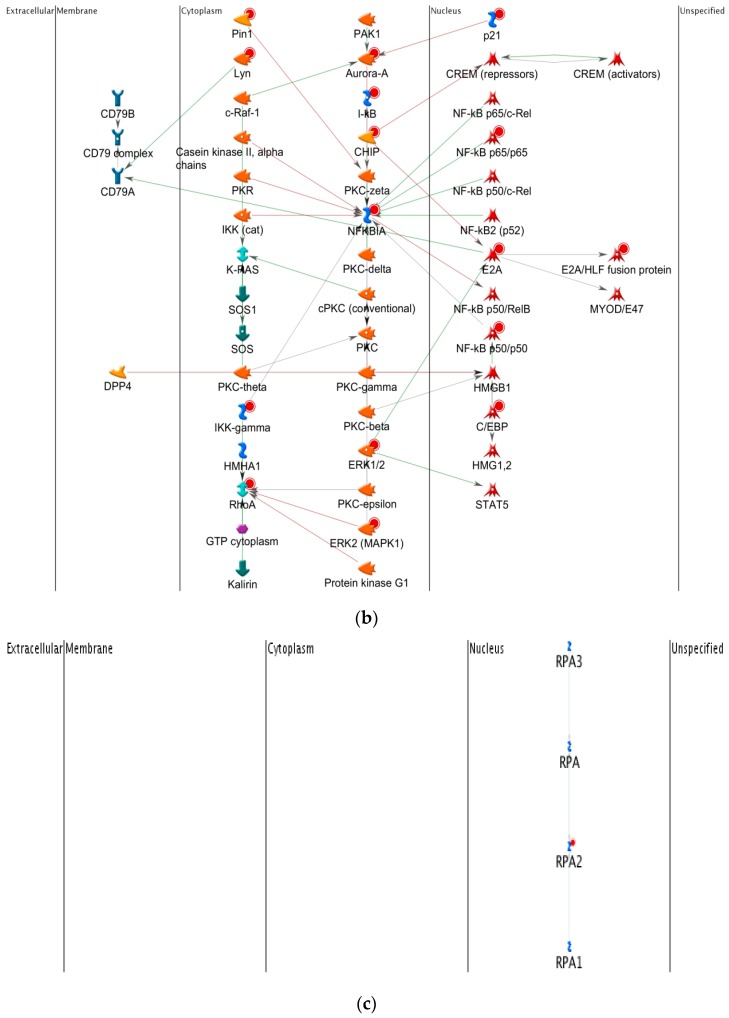

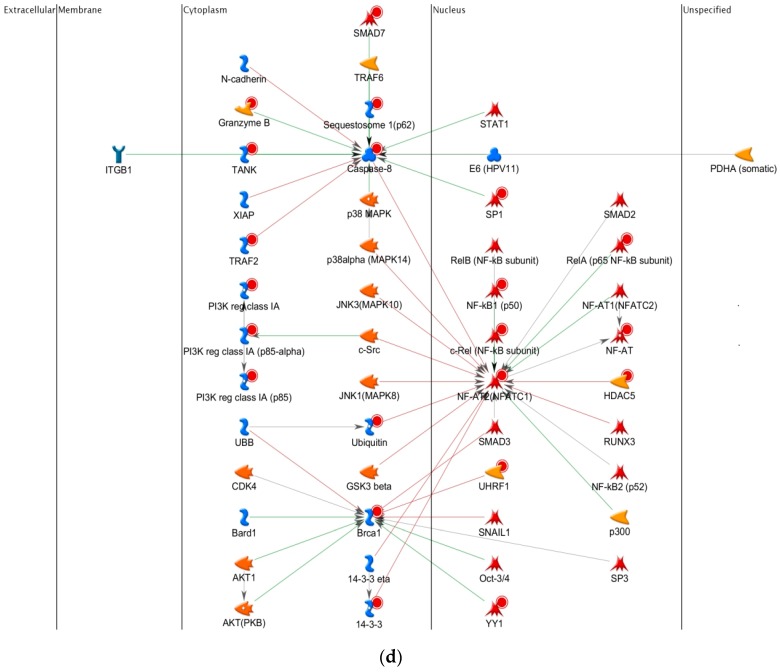
Top-scoring networks for (**a**) 4 h; (**b**) 8 h; (**c**) 24 h; and (**d**) 72 h post-TBI. Scoring was based on the number of pathways. Thick cyan lines indicate fragments of canonical pathways. Up-regulated genes are marked with red circles, and down-regulated with blue circles. A “checkerboard” pattern indicates mixed expression for the gene between files or between multiple tags for the same gene. These figures were generated by MetaCore™.

The AN results for the four time points provide information about canonical pathways, up-regulated and down-regulated genes, and mixed expression genes (Figure 6).

#### 2.4.7. Discussion of the Cell Cycle Behavior of TBI

Our results show that the cell cycle is strongly related to brain injury (Figure 1). Wu *et al.* [30] discuss cell cycle activation and cord injury, stating that a traumatic spinal cord injury (SCI) causes a series of events involving initial mechanical damage, secondary injury processes, and eventually results in tissue loss and functional impairment. They also found that the cell cycle is activated following an SCI [30].

### 2.5. Network Ontology Analysis (NOA) Results

The detailed analytical results of the network ontology analysis (NOA) highlight the evolutionary process at the four time points post-TBI with respect to biological processes, cellular components, and molecular functions (Table 4). For example, the most statistically significant biological process at 4 h post-TBI was protein modification by small-protein conjugation, while the top processes at the other three time points were positive regulation of cellular metabolic processes. The top cellular components at the four time points were protein complex, nucleus, intracellular part, and cytoplasm, respectively. The top molecular function at all four time points was protein binding, followed by enzyme binding, promoter binding, receptor signaling protein activity, and identical protein binding, at 4, 8, 24, and 72 h post-TBI, respectively.

### 2.6. Summary of the Results in Table 4

In addition to our original analysis that identified the PPINs and their corresponding network biomarkers, we have generated abundant results using DAVID (The Database for Annotation, Visualization and Integrated Discovery; Available online: https://david.ncifcrf.gov/home.jsp), MetaCore™, and NOA. Based on our previous experience with cancer research, we applied the David analysis to the four time points post-TBI and expected it to yield the results of KEGG (Kyoto Encyclopedia of Genes and Genomes; Available online: http://www.genome.jp/kegg/). However, we did not find the KEGG database suitable for analyzing TBI (most entries were linked to cancer-related pathways), so we present these results in Supplementary Material S4 and do not describe them here.

Here, we emphasize the importance of the results of the MetaCore™ and NOA analyses. They can be used as references for medical and basic researchers performing further explorations of the hidden mechanisms of network biomarkers, and will also help these researchers to develop novel strategies for TBI therapy or recovery processes. Our original results can also be used for further analysis using other methods, so we present them here.

**Table 4:** Network ontology pathway analysis and gene set enrichment analysis for four time points post-TBI with respect to (1) biological processes; (2) cellular components; and (3) molecular functions. Each of these tables is presented for each of the four time points. R: number of genes in reference set; T: number of genes in test set; G: number of genes annotated by given term in reference set; O: number of genes annotated by given term in test set. This table is produced based on data stored in the network ontology analysis (NOA) web server.

GO: Term	*p-*Value	Corrected *p*-Value	R	T	G	O	Term Name
**4 h Post-TBI**							
**(1) Biological Processes**							
GO:0032446	1.1 × 10^−8^	1.0 × 10^−5^	14,791	26	168	7	protein modification by small protein conjugation
GO:0070647	4.5 × 10^−8^	3.8 × 10^−5^	14,791	26	204	7	protein modification by small protein conjugation or removal
GO:0043687	3.7 × 10^−7^	3.1 × 10^−4^	14,791	26	1243	12	post-translational protein modification
GO:0048518	5.1 × 10^−7^	4.4 × 10^−4^	14,791	26	2188	15	positive regulation of biological process
GO:0043067	8.5 × 10^−7^	7.2 × 10^−4^	14,791	26	850	10	regulation of programmed cell death
GO:0010941	9.0 × 10^−7^	7.7 × 10^−4^	14,791	26	856	10	regulation of cell death
GO:0048522	1.1 × 10^−6^	9.8 × 10^−4^	14,791	26	1984	14	positive regulation of cellular process
GO:0042221	1.3 × 10^−6^	0.0011	14,791	26	1402	12	response to chemical stimulus
GO:0009314	1.5 × 10^−6^	0.0013	14,791	26	215	6	response to radiation
GO:0006464	2.6 × 10^−6^	0.0022	14,791	26	1490	12	protein modification process
**(2) Cellular Components**							
GO:0043234	7.1 × 10^−6^	9.3 × 10^−4^	16,768	25	2748	14	protein complex
GO:0044451	1.8 × 10^−5^	0.0024	16,768	25	594	7	nucleoplasm part
GO:0005737	2.3 × 10^−5^	0.0030	16,768	25	4549	17	cytoplasm
GO:0017053	3.4 × 10^−5^	0.0045	16,768	25	43	3	transcriptional repressor complex
GO:0044428	4.4 × 10^−5^	0.0057	16,768	25	1932	11	nuclear part
GO:0016235	4.4 × 10^−5^	0.0058	16,768	25	7	2	aggresome
GO:0032991	6.5 × 10^−5^	0.0085	16,768	25	3312	14	macromolecular complex
GO:0035098	7.6 × 10^−5^	0.0100	16,768	25	9	2	ESC/E(Z) complex (Extra Sex Combs/Enhancer of Zeste complex)
GO:0034708	7.7 × 10^−5^	0.0100	16,768	25	56	3	methyltransferase complex
GO:0035097	7.7 × 10^−5^	0.0100	16,768	25	56	3	histone methyltransferase complex
**(3) Molecular Functions**							
GO:0005515	7.5 × 10^−7^	1.2 × 10^−4^	15,767	26	8097	25	protein binding
GO:0019899	2.9 × 10^−6^	4.8 × 10^−4^	15,767	26	584	8	enzyme binding
GO:0050815	1.5 × 10^−5^	0.0025	15,767	26	4	2	phosphoserine binding
GO:0032403	4.2 × 10^−5^	0.0069	15,767	26	243	5	protein complex binding
GO:0016563	4.9 × 10^−5^	0.0082	15,767	26	419	6	transcription activator activity
GO:0042802	8.3 × 10^−5^	0.0139	15,767	26	677	7	identical protein binding
GO:0016564	1.7 × 10^−4^	0.0292	15,767	26	329	5	transcription repressor activity
GO:0045309	2.7 × 10^−4^	0.0449	15,767	26	15	2	protein phosphorylated amino acid binding
GO:0004407	3.0 × 10^−4^	0.0513	15,767	26	16	2	histone deacetylase activity
GO:0033558	3.5 × 10^−4^	0.0581	15,767	26	17	2	protein deacetylase activity
**8 h Post-TBI**							
**(1) Biological Processes**							
GO:0031325	4.7 × 10^−11^	5.9 × 10^−8^	14,791	48	965	19	positive regulation of cellular metabolic process
GO:0009893	1.1 × 10^−10^	1.4 × 10^−7^	14,791	48	1015	19	positive regulation of metabolic process
GO:0048523	1.9 × 10^−10^	2.4 × 10^−7^	14,791	48	1815	24	negative regulation of cellular process
GO:0042127	4.4 × 10^−10^	5.6 × 10^−7^	14,791	48	839	17	regulation of cell proliferation
GO:0031328	5.0 × 10^−10^	6.3 × 10^−7^	14,791	48	727	16	positive regulation of cellular biosynthetic process
GO:0009891	6.5 × 10^−10^	8.2 × 10^−7^	14,791	48	740	16	positive regulation of biosynthetic process
GO:0048519	1.2 × 10^−9^	1.5 × 10^−6^	14,791	48	1983	24	negative regulation of biological process
GO:0048518	1.5 × 10^−9^	1.8 × 10^−6^	14,791	48	2188	25	positive regulation of biological process
GO:0051173	2.0 × 10^−9^	2.6 × 10^−6^	14,791	48	683	15	positive regulation of nitrogen compound metabolic process
GO:0031323	2.2 × 10^−9^	2.8 × 10^−6^	14,791	48	3768	32	regulation of cellular metabolic process
**(2) Cellular Components**							
GO:0005634	4.9 × 10^−11^	8.3 × 10^−9^	16768	45	5037	35	nucleus
GO:0044428	7.5 × 10^−11^	1.2 × 10^−8^	16,768	45	1932	23	nuclear part
GO:0032991	1.1 × 10^−7^	1.8 × 10^−5^	16,768	45	3312	25	macromolecular complex
GO:0005737	1.8 × 10^−7^	3.0 × 10^−5^	16,768	45	4549	29	cytoplasm
GO:0043231	3.3 × 10^−7^	5.6 × 10^−5^	16,768	45	7996	38	intracellular membrane-bounded organelle
GO:0043227	3.3 × 10^−7^	5.6 × 10^−5^	16,768	45	7998	38	membrane-bounded organelle
GO:0005829	5.5 × 10^−7^	9.2 × 10^−5^	16,768	45	1269	15	cytosol
GO:0043229	1.1 × 10^−6^	1.8 × 10^−4^	16,768	45	8759	39	intracellular organelle
GO:0043226	1.1 × 10^−6^	1.9 × 10^−4^	16,768	45	8773	39	organelle
GO:0044424	1.6 × 10^−6^	2.8 × 10^−4^	16,768	45	11,001	43	intracellular part
**(3) Molecular Functions**							
GO:0005515	1.2 × 10^−11^	3.1 × 10^−9^	15,767	48	8097	46	protein binding
GO:0010843	4.0 × 10^−11^	9.9 × 10^−9^	15,767	48	111	9	promoter binding
GO:0044212	5.6 × 10^−11^	1.3 × 10^−8^	15,767	48	115	9	DNA regulatory region binding
GO:0016563	4.1 × 10^−7^	1.0 × 10^−4^	15,767	48	419	10	transcription activator activity
GO:0042802	5.9 × 10^−7^	1.4 × 10^−4^	15,767	48	677	12	identical protein binding
GO:0003690	6.2 × 10^−7^	1.5 × 10^−4^	15,767	48	102	6	double-stranded DNA binding
GO:0035326	5.7 × 10^−6^	0.0014	15,767	48	39	4	enhancer binding
GO:0003705	5.7 × 10^−6^	0.0014	15,767	48	39	4	RNA polymerase II transcription factor activity, enhancer binding
GO:0043566	6.1 × 10^−6^	0.0015	15,767	48	151	6	structure-specific DNA binding
GO:0032403	8.1 × 10^−6^	0.0019	15,767	48	243	7	protein complex binding
**24 h Post-TBI**							
**(1) Biological Processes**							
GO:0048522	4.0 × 10^−10^	4.8 × 10^−7^	14,791	46	1984	24	positive regulation of cellular process
GO:0048518	4.5 × 10^−10^	5.4 × 10^−7^	14,791	46	2188	25	positive regulation of biological process
GO:0009987	9.8 × 10^−9^	1.1 × 10^−5^	14,791	46	9216	45	cellular process
GO:0050794	1.3 × 10^−8^	1.5 × 10^−5^	14,791	46	6896	40	regulation of cellular process
GO:0031325	1.4 × 10^−8^	1.7 × 10^−5^	14,791	46	965	16	positive regulation of cellular metabolic process
GO:0051716	1.9 × 10^−8^	2.3 × 10^−5^	14,791	46	847	15	cellular response to stimulus
GO:0048523	2.1 × 10^−8^	2.5 × 10^−5^	14,791	46	1815	21	negative regulation of cellular process
GO:0009893	3.0 × 10^−8^	3.5 × 10^−5^	14,791	46	1015	16	positive regulation of metabolic process
GO:0044260	4.7 × 10^−8^	5.6 × 10^−5^	14,791	46	3428	28	cellular macromolecule metabolic process
GO:0051128	4.9 × 10^−8^	5.9 × 10^−5^	14,791	46	529	12	regulation of cellular component organization
**(2) Cellular Components**							
GO:0044424	5.6 × 10^−9^	1.0 × 10^−6^	16,768	45	11,001	45	intracellular part
GO:0005829	1.1 × 10^−8^	2.1 × 10^−6^	16,768	45	1269	17	cytosol
GO:0005737	1.8 × 10^−7^	3.4 × 10^−5^	16,768	45	4549	29	cytoplasm
GO:0043234	4.1 × 10^−7^	7.9 × 10^−5^	16,768	45	2748	22	protein complex
GO:0005634	4.2 × 10^−7^	8.0 × 10^−5^	16,768	45	5037	30	nucleus
GO:0032991	5.5 × 10^−7^	1.0 × 10^−4^	16,768	45	3312	24	macromolecular complex
GO:0044428	4.7 × 10^−6^	9.1 × 10^−4^	16,768	45	1932	17	nuclear part
GO:0000307	6.4 × 10^−6^	0.0012	16,768	45	14	3	cyclin-dependent protein kinase holoenzyme complex
GO:0070435	7.0 × 10^−6^	0.0013	16,768	45	2	2	Shc-EGFR complex (Src homology 2 domain containing transforming protein-epidermal growth factor receptor complex)
GO:0043229	2.6 × 10^−5^	0.0051	16,768	45	8759	37	intracellular organelle
**(3) Molecular Functions**							
GO:0005515	6.4 × 10^−10^	1.7 × 10^−7^	15,767	46	8097	43	protein binding
GO:0005057	4.0 × 10^−7^	1.1 × 10^−4^	15,767	46	162	7	receptor signaling protein activity
GO:0043560	5.0 × 10^−6^	0.0013	15,767	46	12	3	insulin receptor substrate binding
GO:0019899	5.4 × 10^−6^	0.0015	15,767	46	584	10	enzyme binding
GO:0032403	6.0 × 10^−6^	0.0016	15,767	46	243	7	protein complex binding
GO:0004710	8.3 × 10^−6^	0.0022	15,767	46	2	2	MAP/ERK kinase kinase activity (mitogen-activated protein kinases/extracellular signal-regulated kinases activity)
GO:0045309	1.0 × 10^−5^	0.0028	15,767	46	15	3	protein phosphorylated amino acid binding
GO:0019900	2.4 × 10^−5^	0.0067	15,767	46	201	6	kinase binding
GO:0005488	3.0 × 10^−5^	0.0083	15,767	46	12,581	46	binding
GO:0004702	3.1 × 10^−5^	0.0086	15,767	46	62	4	receptor signaling protein serine/threonine kinase activity
**72 h Post-TBI**							
**(1) Biological Processes**							
GO:0048522	1.60 × 10^−17^	2.50 × 10^−14^	1.48 × 10^4^	56	1984	35	positive regulation of cellular process
GO:0048518	3.80 × 10^−17^	5.60 × 10^−14^	1.48 × 10^4^	56	2188	36	positive regulation of biological process
GO:0031325	1.60 × 10^−12^	2.40 × 10^−09^	1.48 × 10^4^	56	965	22	positive regulation of cellular metabolic process
GO:0009893	4.50 × 10^−12^	6.70 × 10^−09^	1.48 × 10^4^	56	1015	22	positive regulation of metabolic process
GO:0010604	9.20 × 10^−12^	1.30 × 10^−08^	1.48 × 10^4^	56	939	21	positive regulation of macromolecule metabolic process
GO:0032502	9.30 × 10^−12^	1.30 × 10^−08^	1.48 × 10^4^	56	3032	35	developmental process
GO:0048519	5.20 × 10^−11^	7.70 × 10^−08^	1.48 × 10^4^	56	1983	28	negative regulation of biological process
GO:0006950	1.00 × 10^−10^	1.50 × 10^−07^	1.48 × 10^4^	56	1591	25	response to stress
GO:0042981	1.00 × 10^−10^	1.50 × 10^−07^	1.48 × 10^4^	56	842	19	regulation of apoptosis
GO:0010033	1.20 × 10^−10^	1.80 × 10^−07^	1.48 × 10^4^	56	850	19	response to organic substance
**(2) Cellular Components**							
GO:0005737	9.2 × 10^−11^	1.9 × 10^−8^	16,768	57	4549	39	cytoplasm
GO:0043234	1.8 × 10^−9^	4.0 × 10^−7^	16,768	57	2748	29	protein complex
GO:0005829	2.6 × 10^−9^	5.6 × 10^−7^	16,768	57	1269	20	cytosol
GO:0032991	6.7 × 10^−9^	1.4 × 10^−6^	16,768	57	3312	31	macromolecular complex
GO:0044428	2.4 × 10^−8^	5.2 × 10^−6^	16,768	57	1932	23	nuclear part
GO:0044424	1.6 × 10^−7^	3.5 × 10^−5^	16,768	57	11,001	54	intracellular part
GO:0044446	2.0 × 10^−7^	4.4 × 10^−5^	16,768	57	5015	36	intracellular organelle part
GO:0044422	2.9 × 10^−7^	6.3 × 10^−5^	16,768	57	5082	36	organelle part
GO:0005634	9.0 × 10^−7^	1.9 × 10^−4^	16,768	57	5037	35	nucleus
GO:0070435	1.1 × 10^−5^	0.0024	16,768	57	2	2	Shc-EGFR complex
**(3) Molecular Functions**							
GO:0005515	2.8 × 10^−17^	6.5 × 10^−15^	15,767	57	8097	57	protein binding
GO:0042802	8.7 × 10^−8^	1.9 × 10^−5^	15,767	57	677	14	identical protein binding
GO:0019899	1.1 × 10^−7^	2.6 × 10^−5^	15,767	57	584	13	enzyme binding
GO:0032403	2.4 × 10^−6^	5.5 × 10^−4^	15,767	57	243	8	protein complex binding
GO:0005488	2.5 × 10^−6^	5.7 × 10^−4^	15,767	57	12,581	57	binding
GO:0019900	7.5 × 10^−6^	0.0017	15,767	57	201	7	kinase binding
GO:0016566	1.0 × 10^−5^	0.0023	15,767	57	38	4	specific transcriptional repressor activity
GO:0019901	3.2 × 10^−5^	0.0072	15,767	57	169	6	protein kinase binding
GO:0010843	4.9 × 10^−5^	0.0112	15,767	57	111	5	promoter binding
GO:0044212	5.8 × 10^−5^	0.0132	15,767	57	115	5	DNA regulatory region binding

## 3. Experimental Section

### 3.1. Overview of the Traumatic Brain Injury (TBI) Network Biomarkers Identification Process

**Figure 7:**
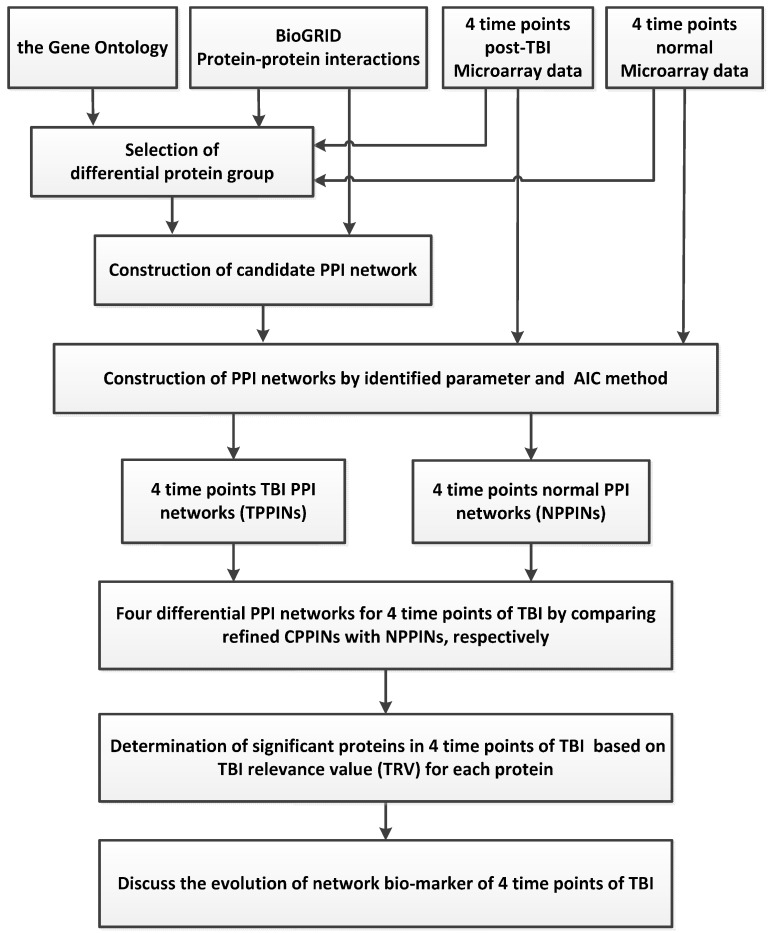
The method by which we constructed the network markers for four time points with respect to TBI and investigated the underlying molecular mechanisms of TBI. BioGRID: Biological General Repository for Interaction Database.

We used a theoretical framework to identify the evolution of TBI network biomarkers at four time points representing four important stages after the occurrence of a TBI. We have successfully used a similar theoretical framework to identify the network biomarkers of various cancers and stroke [31,32,33]. We therefore highlight only the key points of this framework in this section, and describe the process in detail in the supplementary information (Supplementary Material S0). The flowchart in Figure 7 illustrates how we identified the network biomarkers of TBI at four time points. We combined two kinds of data sets to develop our model: (i) microarray data from both TBI and normal samples from the Gene Expression Omnibus (GEO) database, where TBI samples are divided into four groups according to the time post-injury (4, 8, 24, and 72 h); and (ii) a protein–protein interaction database, which was necessary to construct four candidate PPINs for TBI.

Using several mathematical models, we constructed four TBI PPINs (TPPINs) and a normal PPIN (NPPIN). We also constructed four Differential PPINs (DPPINs) and calculated the TBI relevance value (TRV) for each protein in the DPPINs (Supplementary Material S0).

### 3.2. Data Selection and Pre-Processing

To identify the regulatory mechanisms of brain trauma, we obtained microarray datasets from gene expression profiling of brain injury samples (4, 8, 24, and 72 h after surgery) and brain sham samples (4, 8, 24, and 72 h after sham surgery) in mice from the NCBI GEO [34]. We chose GSE2392 [35] and its corresponding platform, GPL81, as our research object (Table 1). We collected expression profiling data for mice from the Gene Expression Omnibus (GEO), accession number GSE2392, and normalized it using quantile normalization. For each of the four time points, there were three TBI samples and two control samples, making a total of 20 samples.

The PPI dataset for *Mice* was downloaded from the public Biological General Repository for Interaction Database (BioGRID) [36]. We excluded false-positive PPIs from the TBI PPINs using BioGRID and compared the resulting PPINs to identify network biomarkers (Supplementary Material S0).

### 3.3. Selection of the Differential Protein Groups and Identification of PPINs

We combined the gene expression profiles with the PPI database to construct the corresponding TPPINs and NPPIN. First, we identified a differential protein group containing proteins with differential expression. Protein expression profiles are not yet readily available, so we assumed that gene expression profiles are correlated with protein expression profiles. We used a one-way analysis of variance (ANOVA) or the fold change (FC) method to analyze the expression of each protein and select the proteins that were differentially expressed in cells. These methods were capable of identifying the critical proteins differentially expressed in TBI and normal samples. We eliminated proteins with no PPI information from the differential protein group. Proteins that were not already in the differential protein group were included if their PPI information indicated that they had a close relationship with a protein (number of edges > 5) in the group. Thus, the differential protein group included the critical differential expression proteins and other closely related proteins.

We combined the critical differential protein group and PPI information to construct candidate PPINs by linking proteins that interacted with each other. In other words, proteins for which we had PPI information via the differential protein group were linked using our mathematical model to form the candidate TPPINs and NPPINs.

The candidate TPPINs and NPPINs included all possible PPIs under various biological conditions. It was then necessary to confirm them using microarray expression data and identify the proper PPIs according to the TBI processes. We used both the PPI model selection method and a model order detection strategy to exclude the false-positive PPIs. We could then express the relationship to the PPI of the *i*-th target protein in the candidate TPPINs and NPPINs with the following formula:

(1) $$x_{i}(n)=\sum_{j=1}^{M_{i}}\alpha_{ij}x_{j}(n)+\omega_{i}(n)$$

where *x_i_*(*n*) is the expression levels of target protein *i* for sample *n*; *x_j_*(*n*) is the expression level of the *j*-th protein interacting with target protein *i* for sample *n*; α*_ij_* is the association ability between target protein *i* and its *j*-th interactive protein; *M_i_* is the number of proteins interacting with target protein *i*; and ω*_i_*(*n*) is stochastic noise due to other factors or model uncertainty. For the definitions of the terms, please refer to Supplementary Material S1.

We then used the least squares parameter estimation method [37] to determine the parameter of association strength in Equation (1) from the TBI microarray data as follows (Supplementary Material S1):

(2) $$x_{i}(n)=\sum_{j=1}^{M_{i}}{\hat{\alpha }}_{ij}x_{j}(n),i=1,2,\cdot \cdot \cdot ,M$$

where ${\hat{\alpha }}_{ij}$ is solved using TBI microarray data and the least squares parameter estimation method.

Finally, we use AIC (Akaike information criterion) [37] with a Student’s *t*-test [38] for model order determination and to determine the critical protein association strengths ${\hat{\alpha }}_{ij}$ (Supplementary Material S2).

### 3.4. Determination of Proteins with Top TBI Relevance Value (TRV)and Their Corresponding Network Structures

The significant remaining PPIs after pruning the false-positives are expressed by the following:

(3) $$x_{i}(n)=\sum_{j=1}^{{M_{i}}^{\prime }}{\hat{\alpha }}_{ij}x_{j}(n)+w_{i}^{\prime }(n),i=1,2,\cdot \cdot \cdot ,M$$

where *M_i_*′ ≤ *M_i_* is the total number of significant PPIs remaining in the refined PPINs for target protein *i*. The final refined PPIN is as follows:

(4) X(n)=AX(n)+w(n)

$$X(n)=\left[\begin{array}{c} x_{1}(n)\\ x_{2}(n)\\ \vdots \\ x_{M}(n) \end{array}\right],A=\left[\begin{array}{ccc} {\hat{\alpha }}_{11} & \cdots & {\hat{\alpha }}_{1M}\\ \vdots & \ddots & \vdots \\ {\hat{\alpha }}_{M1} & \cdots & {\hat{\alpha }}_{MM} \end{array}\right],w(n)=\left[\begin{array}{c} w_{1}^{\prime }(n)\\ w_{1}^{\prime }(n)\\ \vdots \\ w_{M}^{\prime }(n) \end{array}\right]$$

The interaction matrix *A* of refined PPINs (*i.e.*, after pruning, as described by Equation (4) were constructed as follows:

(5) $$A_{T}^{K}=\left[\begin{array}{ccc} {\hat{\alpha }}_{11,T}^{k} & \cdots & {\hat{\alpha }}_{1M,T}^{k}\\ \vdots & \ddots & \vdots \\ {\hat{\alpha }}_{M1,T}^{k} & \cdots & {\hat{\alpha }}_{MM,T}^{k} \end{array}\right]A_{N}=\left[\begin{array}{ccc} {\hat{\alpha }}_{11,N} & \cdots & {\hat{\alpha }}_{1M,N}\\ \vdots & \ddots & \vdots \\ {\hat{\alpha }}_{M1,N} & \cdots & {\hat{\alpha }}_{MM,N} \end{array}\right]$$

where *k* is time post-injury, $A_{T}^{K}$ and *A_N_* are the interaction strength matrices of the refined TPPINs and NPPIN for 4, 8, 24, and 72 h post-injury, respectively, and *M* is the total number of interaction proteins in the refined TPPINs and NPPIN after pruning. Thus, the protein interaction model can be expressed as follows:

(6) $$\begin{array}{l} x_{T}^{K}(n)=A_{T}^{k}x_{T}(n)\\ x_{N}(n)=A_{N}x_{N}(n) \end{array}$$

where *k* is time post-injury and $x_{T}^{k}(n)={\left[x_{1T}^{k}\;x_{2T}^{k}\;\cdots x_{MT}^{k}\right]}^{T}$ and $x_{N}(n)={\left[x_{1N}\;x_{2N}\;\cdots x_{MN}\right]}^{T}$ are vectors of protein expression values. The difference matrix *D^k^* we used to interpret the differences in behavior between two networks is defined as follows:

(7) $$D^{k}=\left[\begin{array}{ccc} d_{11}^{k} & \cdots & d_{1M}^{k}\\ \vdots & \ddots & \vdots \\ d_{M1}^{k} & \cdots & d_{MM}^{k} \end{array}\right]=\left[\begin{array}{ccc} {\hat{\alpha }}_{11,T}^{k}-{\hat{\alpha }}_{11,N} & \cdots & {\hat{\alpha }}_{1M,T}^{k}-{\hat{\alpha }}_{1M,N}\\ \vdots & \ddots & \vdots \\ {\hat{\alpha }}_{M1,T}^{k}-{\hat{\alpha }}_{M1,N} & \cdots & {\hat{\alpha }}_{MM,T}^{k}-{\hat{\alpha }}_{MM,N} \end{array}\right]$$

where *k* is time post-injury, $d_{ij}^{k}$ is the difference between the protein association capacity of the networks at different times post-injury, and matrix *D^k^* denotes the differences in network structures. To investigate TBI-related factors using matrix *D^k^*, we proposed a value we called the TBI relevance value (TRV) to quantify the significance of each protein in *D^k^* as follows [13]:

(8) $$TRV^{k}=\left[\begin{array}{c} TRV_{1}^{k}\\ \vdots \\ TRV_{i}^{k}\\ \vdots \\ TRV_{M}^{k} \end{array}\right]$$

where $TRV_{i}^{k}=\sum_{j=1}^{M}\left|d_{ij}^{k}\right|$, and *k* is time post-injury (Supplementary Material S0).

### 3.5. Pathway Analysis

Our theory allowed us to construct the TPPINs and NPPINs and identify the network biomarkers with the highest TRVs. However, we also wanted to explore the biological significance of these network biomarkers; here, we used various software packages and on-line tools to do the functional pathway analysis. For example, we used KEGG [39], the DAVID bioinformatics database [40,41], and NOA [42,43], which are the most commonly used free on-line tools, and MetaCore™ from Thomson Reuters, a powerful commercial software package that can do multiple functional and pathway analyses. A detailed description of the process we followed is given in Supplementary Material S0.

### 3.6. Software and Databases Used in This Research

We conducted our analyses (AIC, student’s t-test and least squares) using MATLAB’s built-in functions. We also constructed the TPPINs, NPPIN, and TRB using MATLAB (The MathWorks, Inc., Natick, MA, USA). The version of the programs we used is not yet open-access. However, we suggest that readers refer to the old version of the theoretical framework and source code, which are available to the public [44] (Supplementary Material S3).

We used the MetaCore™ Enrichment Analysis (EA) Workflow tool (Thomson Reuters, New York, NY, USA) to analyze the experimental data in terms of their enrichment by the various ontologies in MetaCore™, including ontologies and GO ontologies: Pathway Maps, process networks, Diseases, metabolic networks, toxicity networks, and GO processes. The EA method involves mapping the selected experiments onto MetaCore™ ontologies in terms of their respective sets of genes or network objects, which are then sorted according to their *p-*values. We used the Compare Experiments Workflow tool (GeneGO, Inc., Encinitas, CA, USA) to compare the experimental data for the four time points post-TBI by analyzing their intersections in terms of their mapping onto the MetaCore™ ontologies. The Experiment Intersections method involves mapping the intersections of the selected experiments, which identifies the sets of genes common to all selected experiments, similar to some of them, or unique to a given experiment. We collated all of these sets of genes for further analysis and discussion. MetaCore™ is user-friendly, and various manuals and training materials can be download from public websites [45,46]. However, due to copyright concerns, we have not copied the material here. We used Cytoscape Web [47] to display the networks of critical biomarkers. Nodes and edges in the network represent genes and expression correlations between two genes, respectively. We used the ForceDirected approach as the default layout. Users can change the network display settings by altering parameter settings for nodes, edges, and layout. The GO database holds both the ontologies and the annotations in a single database and allows queries of the annotations and enrichment analyses using the ontologies [48]. DAVID, KEGG, and NOA (A cytoscape plugin for network ontology analysis; Available online: http://apps.cytoscape.org/apps/noa) are easy-to-use web servers with user-friendly interfaces.

## 4. Conclusions

In conclusion, we used a publicly available high-throughput microarray to construct networks for four time points post-TBI. The powerful functional pathway analysis then extended the relevance of our research to a wider field. This work can help scientists and medical researchers reveal the underlying hidden mechanisms of TBI and improve biological models for TBI. However, the number of samples in this study was small. In future, we will include more data samples from humans and model organisms to develop a more precise and accurate model. In addition, we will integrate the GRN and PPI models to facilitate deeper interpretation of the regulatory and interaction mechanisms. This work can offer clues and information for developing novel biological models and strategies for target therapy to advance recovery processes.

## Supplementary Materials

Supplementary materials can be found at http://www.mdpi.com/1422-0067/17/2/216/s1.
