# Supplementary material for: Temporal Genetic Modifications after Controlled Cortical Impact—Understanding Traumatic Brain Injury through a Systematic Network Approach
Source: Int J Mol Sci. 2016 Feb 6;17(2):216. doi: 10.3390/ijms17020216 (PMC4783948; doi:10.3390/ijms17020216)
Supplement: Supplementary file 1 [file ijms-17-00216-s001.zip › ijms-105508-Supplementary Materials/ijms-105508-supplementary.pdf]

Supplementary Material: Temporal Genetic Modifications after Controlled Cortical Impact—Understanding Traumatic Brain Injury through a Systematic Network Approach

Yung-Hao Wong, Chia-Chou Wu, John Chung-Che Wu, Hsien-Yong Lai, Kai-Yun Chen, Bo-Ren Jheng, Mien-Cheng Chen, Tzu-Hao Chang and Bor-Sen Chen

S0. Detailed Description of Material and Methods

1.1. Overview of the Traumatic Brain Injury (TBI) Network Biomarkers Identification Process

A theoretical framework was employed in this study to find the evolution of TBI network biomarkers at four time points which represent four important stages after the occurrence of TBI [31,32]. A flowchart to identify network biomarkers of TBI at four time points is shown in Figure 7 in main text. We combined two kinds of data sources: (i) microarray data of TBI and normal samples from the Gene Expression Omnibus (GEO) database where TBI samples are divided into four groups according to the time points of 4, 8, 24, and 72 h post-injury; and (ii) a PPI database, which was required to construct four candidate PPINs of TBI.

Microarray data were used for PPI pool selection, and then the selected PPIs and expression profiles of differentially expressed genes were used to construct a PPIN. Through regression modeling and a least square parameter estimation method, four TBI PPINs (TPPINs) and a normal PPIN (NPPIN) were then obtained. We also created four Differential PPINs (DPPINs) by comparing the constructed TPPINs with the NPPIN. In DPPINs, a set of significant proteins for TBI were obtained as network biomarkers at each time point (stage) based on the TBI relevance value (TRV) of each protein in Equation (8) and a statistical assessment stated in subsection D of Materials and Methods section.

We integrated the GO database, and protein–protein interaction (PPI) information to construct the PPI network of Brain injury. These data were used for pool selection, and then the selected proteins were used to contribute to the PPI network (PPIN) by a maximum-likelihood estimation and model order detection method, resulting in a TBI PPIN (TPPIN) and a normal PPIN (NPPIN) in the early and late stages of brain injury. The two constructed PPINs were used to determine significant proteins of tumorigenesis by examining differences between the two PPI matrices of the two constructed PPINs. With the help of a differential PPI matrix (network) between CPPIN and NPPIN, a TBI relevance value (TRV) was computed for each protein, and significant proteins in carcinogenesis were determined based on p values of the TRVs of these proteins in the differential PPI matrix between CPPIN and NPPIN.

1.2. Data Selection and Pre-Processing

To identify the potential molecular mechanisms of brain trauma, the microarray dataset of the gene expression profiling data of lateral fluid percussion-induced brain injury and lateral controlled cortical impact injury in mice and normal samples was obtained from the NCBI GEO [34]. In this study, we chose GSE2392 [35] and its corresponding platform, GPL81, as our research object (Table 1). It contains gene expression data following a TBI. The expression profiling data of mice were collected from Gene Expression Omnibus (GEO) with the accession number of GSE2392, and normalized by quantile normalization.

Brain injury samples were generated from male C57BL/6 mice (20–25 g) which were anesthetized with isoflurane/oxygen. Moderate controlled cortical impact with pneumatic impactor, 6 m/s impact velocity and 1.5 mm deformation. 4, 8, 24 and 72 h after surgery, a 4 mm diameter disk of parieto-occipital cortex, centered on the point of impact of the injury and containing the contusion area, was removed and immediately frozen in chilled 2-methylbutane then stored at

−80 °C. Three mouse cortex disks were pooled prior to RNA extraction. Brain sham samples were generated form male Sprague-Dawley rats (400 ± 25 g) which were anesthetized with sodium pentobarbital. 4, 8, 24, 72 h after sham surgery, a 7 mm disk of parieto-occipital cortex was removed and immediately frozen in chilled 2-methylbutane and then stored at·−80 °C. Cortical tissue from one rat provided sufficient RNA without the need for pooling [35].

We only used data derived from non-processed primary biopsies to avoid discrepancies in gene expressions that are intrinsic to cell culture and fixation. Therefore, the dataset contained 4 time points of TBI mice and two control samples from non-disease subjects. We built the TPPINs for 4, 8, 24 and 72 h post-TBI and the NPPIN in this study. The dataset contained three samples for each stage. Prior to further analysis, the gene expression value, h_{ij}, of gene i in the jth sample was normalized to z-transformed scores, g_{ij}, and then the resultant normalized expression value had a mean $\mu_i = 0$ and standard deviation $\sigma_i = 1$ over sample j [13].

PPI data for *Mice* were extracted from the Biological General Repository for Interaction Database (BioGRID). The BioGRID is an open-access archive of genetic and protein interactions that are curated from the primary biomedical literature of all major model organisms [36]. BioGRID was mined for candidate TBI PPINs which were pruned to delete false-positive PPIs using their corresponding microarray data. These PPINs of 4, 8, 24, and 72 h post-injury and normal stages were then compared to obtain network biomarkers.

1.3. Selection of a Protein Pool and Identification of PPINs for Normal and Four TBI Stages

To integrate gene expressions with PPI data for constructing the corresponding TPPINs and NPPIN, we first set up a protein pool containing differentially expressed proteins (DEPs). Owing to the lack of protein expression data, gene expression levels were reasonably assumed to correlate with protein expression levels. We used a one-way analysis of variance (ANOVA) to analyze the expression of each protein and select for proteins with significant differential expression levels. This method allowed determination of significant differentially expressed proteins between TBI and normal samples. However, proteins with no PPI information were eliminated from the protein pool. In addition, proteins that were not already in the protein pool were included if their PPI information indicated that they had a close relationship with a protein (# of edge >5) in the pool. As a result, the protein pool contained the significant DEPs and the proteins that had close relationships with the significant DEPs.

On the strength of the significant protein pool and PPI information, candidate PPINs for 4, 8, 24, and 72 h post-injury and normal stages were constructed by linking proteins that interacted with each other. In other words, proteins that had PPI information through the pool were linked together, resulting in candidate PPIN.

As the candidate PPIN included all possible PPIs under various environmental and experimental conditions, the candidate PPIN is needed to be further confirmed by microarray data and identified appropriate PPIs according to the TBI processes. To remove false-positive PPIs from each candidate PPIN for different biological conditions, we used both a PPI model identification scheme and a model order detection method to prune each candidate PPIN using corresponding microarray data to approach the actual PPIN of TBI. Here, the PPI of target protein i in the candidate PPIN can be depicted by the following protein association model:

$$x_i(n) = \sum_{j=1}^{M_i} \alpha_{ij} x_j \omega_i(n) \tag{1}$$

where $x_i(n)$ is the expression levels of target protein i for sample n; $x_j(n)$ is the expression level of the j-th protein interacting with target protein i for sample n; α_{ij} is the association ability between target protein i and its j-th interactive protein; M_i is the number of proteins interacting with target protein i; and $\omega_i(n)$ is stochastic noise due to other factors or model uncertainty. The biological meaning of Equation (1) is that expression levels of target protein i are associated with expression levels of

proteins that interact with it. Consequently, a protein association model for each protein in the protein pool can be built using Equation (1).

After constructing Equation (1) for the PPI model of each protein in the candidate PPIN, we used the least square parameter estimation method [37] to identify associated parameters in Equation (1) by microarray data as follows (see Additional file S1):

$$x_i(n) = \sum_{j=1}^{M_i} \hat{\alpha}_{ij} x_j(n), \ i = 1, \ 2, \ \cdots, \ M \tag{2}$$

where $\hat{\alpha}_{ij}$ is identified using microarray data with the least square parameter estimation method.

Once the associated parameters for all proteins in the candidate PPIN were identified, significant protein associations were determined using the model order detection method based on the estimated association abilities, *i.e.*, detecting the interaction number, M_i, in Equation (2). The Akaike information criterion (AIC) [37] and a Student's *t*-test [38] were used for both model order selection and significance determination of protein association abilities $\hat{\alpha}_{ij}$ (see Supplementary Material S2).

1.4. Determination of Significant Proteins and Their Network Structures at 4, 8, 24 and 72 h Post-TBI and Normal Samples

After the interaction number, M_i', was determined using the AIC order detection and Student's *t*-test, false-positive PPIs in Equation (2) were pruned away, only significant PPIs were remained, and Equation (2) is refined as follows:

$$x_i(n) = \sum_{j=1}^{M_i'} \hat{\alpha}_{ij} x_j(n), \ i = 1, \ 2, \ \cdots, \ M \tag{3}$$

where $M_i' \le M_i$ is the number of significant PPIs in the refined PPINs, with target protein *i*. In other words, a number of $M_i' \le M_i$ (or false positives) was pruned from the PPIs of target protein *i*. By collecting all proteins in PPIN (*i.e.*, $i = 1, 2, \cdots, M$ for all proteins in Equation (3)), Equation (3) can be further written as follows:

$$X(n) = AX(n) \tag{4}$$

where

$$X(n) = \begin{bmatrix} x_1(n) \\ x_2(n) \\ \vdots \\ x_M(n) \end{bmatrix}, \ A = \begin{bmatrix} \hat{\alpha}_{11} & \cdots & \hat{\alpha}_{1M} \\ \vdots & \ddots & \vdots \\ \hat{\alpha}_{M1} & \cdots & \hat{\alpha}_{MM} \end{bmatrix}$$

The interaction matrix *A* denotes PPIs in their refined PPIN. If there was no PPI between proteins *i* and *j*, it was pruned away by AIC order detection due to insignificance in the refined PPIN and then set $\hat{\alpha}_{ij} = 0$. In general, $\hat{\alpha}_{ij} = \hat{\alpha}_{ji}$. If it is not the case, the larger one is chosen as $\hat{\alpha}_{ij} = \hat{\alpha}_{ji}$ to avoid the situation where $\hat{\alpha}_{ij} \ne \hat{\alpha}_{ji}$. The above PPIN construction method was employed to construct refined PPINs for 4, 8, 24, and 72 h post-injury and normal samples. The interaction matrixes *A* of refined PPINs in Equation (4) for 4, 8, 24, and 72 h post-injury and normal samples were constructed, respectively, as follows:

$$A_N = \begin{bmatrix} \hat{\alpha}_{11,N} & \cdots & \hat{\alpha}_{1M,N} \\ \vdots & \ddots & \vdots \\ \hat{\alpha}_{M1,N} & \cdots & \hat{\alpha}_{MM,N} \end{bmatrix} \tag{5}$$

where k = 4, 8, 24, and 72 h post-injury; A_T^K and A_N are interaction matrices of the refined PPINs of 4, 8, 24, and 72 h post-injury, respectively; and M is the number of proteins in the refined PPIN. Therefore, the protein association model of TPPINs and the NPPIN for 4, 8, 24, and 72 h post-injury and normal samples can be represented by the following equations according to Equations (4) and (5):

$$\begin{aligned} x_T^K(n) &= A_T^k x_T(n) \\ x_N(n) &= A_N x_N(n) \end{aligned} \tag{6}$$

where k = 4, 8, 24, and 72 h post-injury and $x_T^k(n) = [x_{1T}^k \; x_{2T}^k \; \cdots x_{MT}^k]^T$ and $x_N(n) = [x_{1N} \; x_{2N} \; \cdots x_{MN}]^T$ are vectors of protein expression levels. The difference matrix D^k between TPPINs and NPPIN is defined as follows:

$$D^k = \begin{bmatrix} d_{11}^k & \cdots & d_{1M}^k \\ \vdots & \ddots & \vdots \\ d_{M1}^k & \cdots & d_{MM}^k \end{bmatrix} = \begin{bmatrix} \hat{\alpha}_{11,T}^k - \hat{\alpha}_{11,N} & \cdots & \hat{\alpha}_{1M,T}^k - \hat{\alpha}_{1M,N} \\ \vdots & \ddots & \vdots \\ \hat{\alpha}_{M1,T}^k - \hat{\alpha}_{M1,N} & \cdots & \hat{\alpha}_{MM,T}^k - \hat{\alpha}_{MM,N} \end{bmatrix} \tag{7}$$

where k = 4, 8, 24, and 72 h post-injury; d_{ij}^k is the difference between protein association abilities of NPPIN and TPPINs at k = 4, 8, 24, and 72 h post-injury; and matrix D^k is the difference in network structures between TPPINs and the NPPIN for k = 4, 8, 24, and 72 h post-injury and normal samples. In order to investigate TBI-related factors from the difference matrix, D^k, between TPPINs and the NPPIN at 4, 8, 24, and 72 h post-injury and normal cells in Equation (7), a value named as TBI relevance value (TRV), is proposed to quantify the significance of each protein in difference matrix D^k of TBI as follows [6]:

$$TRV^k = \begin{bmatrix} TRV_1^k \\ \vdots \\ TRV_i^k \\ \vdots \\ TRV_M^k \end{bmatrix} \tag{8}$$

where $TRV_i^k = \sum_{j=1}^{M} \left| d_{ij}^k \right|$, and k = 4, 8, 24, and 72 h post-injury. The TRV_i^k in Equation (8) quantifies the differential extent of protein associations of the i-th protein (the absolute sum of the i-th row of D^k in Equation (7)) and the TRV_i^k can differentiate the TPPIN from the NPPIN in the k-th stage of TBI. In other words, the TRV_i^k in Equation (8) represents the network structural difference of the i-th protein between the TPPIN and NPPIN in the k-th stage of TBI.

In order to investigate the likelihoods of proteins involved in the k-th stage of TBI, the corresponding empirical p-value to determine the statistical significance of the TRV_i^k is needed. To determine the empirical p-value of each TRV_i^k, we repeatedly permuted the network structure of the candidate PPINs at 4, 8, 24, and 72 h post-injury as a random network. Each protein in the random network at 4, 8, 24, and 72 h post-injury had its own *TRV* to generate a distribution of TRV_i^k for k = 4, 8, 24, and 72 h post-injury. Although there was a random arrangement of the network structure, linkages of each protein were maintained. In other words, proteins with which a particular protein interacted were permuted without changing the total number of protein interactions. This procedure was repeated 100,000 times and the corresponding p-value was calculated as the fraction of the random network structure in which the TRV_i^k was at least as large

as the *TRV* of the actual network structure. According to the distributions of the TRV_i^k, the TRV_i^k in Equation (8) with a *p*-value of ≤0.01 was regarded as a significant *TRV*, and the corresponding protein was referred as a significant protein at 4, 8, 24, and 72 h post-injury.

Based on the *p*-value of the *TRVs* for all proteins ($i = 1, 2, \cdots, M$) and the 4 stages of TBI (k = 4, 8, 24, and 72 h post-injury), we generated 2 lists of significant proteins for each stage according to the *TRV* distribution and the statistical assessment of each significant protein in TRV_i^k in Equation (8). We also found 27, 50, 48 and 59 significant proteins, respectively, as the specific network biomarkers of 4, 8, 24, and 72 h post-injury. These proteins showed significant changes between TPPINs and the NPPIN in the TBI process according to the corresponding TBI stage, and we speculated that these changes may play important roles in the TBI process. These findings warrant further investigation.

1.5. Pathway Analysis

More-valuable cellular information can be found in known pathways, which are useful for describing most "normal" biological phenomena. All of these known pathways are the result of repeated testing and verification, and the entire pathway network has definitions for most links. This approach supports the view that systems biology can help identify significant network biomarkers in TBI and relate their cellular roles in sTBI etiology and repair processes.

To do the pathway analysis is not a simple work in this research. KEGG [40] and DAVID bioinformatics database [41,42] are the most common tools for such kind analysis. However, they are not so powerful in the case of TBI or stroke, and most of the pathways we found are cancer related.

To complete our research results, we used the well-known commercial software MetaCore from Thomson Reuters to do multiple functional and pathway analyzes. We then used the network ontology analysis (NOA) free software to do the pathway analysis and gene set enrichment analysis (GSEA) on biological processes, cellular components, and molecular functions [43,44]. NOA first defines link ontology that assigns functions to interactions based on the known annotations of joint genes via optimizing two novel indexes "Coverage" and "Diversity". Then, NOA generates two alternative reference sets to statistically rank the enriched functional terms for a given biological network. J. Wang *et al.* compare NOA with traditional enrichment analysis methods in several biological networks, and find that: (i) NOA can capture the change of functions not only in dynamic transcription regulatory networks but also in rewiring protein interaction networks while the traditional methods cannot; and (ii) NOA can find more relevant and specific functions than traditional methods in different types of static networks. The above description of NOA is directly cited from their papers [43].

S1. Parameter Identification of Regression Model in Equation (1) by Maximum Likelihood Method

Equation (1) can be written as the following requiring form

$$x_i[n] = [x_1[n] \cdots x_{Mi}[n]] \begin{bmatrix} a_{i1} \\ \vdots \\ \alpha_{iMi} \end{bmatrix} + \omega_i[n] = \phi_i[n] \cdot \theta_i + \omega_i[n] \tag{S1}$$

where $\phi_i[n]$ denotes the regression vector which can be obtained from microarray data, θ_i is the parameter vector to be estimated. Suppose that there are m samples, then it is easy to acquired values of $\{x_i[n]\phi_i[n]\}$ for $n \in \{1, \ldots, m\}$. In this case, Equation (S1) for different samples can be represented as the following vector form.

$$\begin{bmatrix} x_i[1] \\ \vdots \\ x_i[m] \end{bmatrix} = \begin{bmatrix} \phi_i[1] \\ \vdots \\ \phi_i[m] \end{bmatrix} \cdot \theta_i + \begin{bmatrix} \omega_i[1] \\ \vdots \\ \omega_i[m] \end{bmatrix} \quad \text{(S2)}$$

where $\phi_i[m] = [x_i[m] \cdots x_{Mi}(n)]$, $\theta_i = [\alpha_{i1} \cdots \alpha_{iMi}]$.

For simplicity, it can be represented as follows.

$$X_i = \Phi_i \cdot \theta_i + e_i \quad \text{(S3)}$$

where $e_i = [w_i(1) \cdots w_i(m)]^T$.

In Equation (S3), the noise e_i for different samples was regarded as independent random variables of normal distribution with zero mean and unknown variance σ_i^2, *i.e.*, $E\{e_i\} = 0$, and $\sum_i = E\{e_i e_i^T\} = \sigma_i^2 I$, where I is the identity matrix. The probability density function of e_i is given as follows.

$$p(e_i) = \frac{1}{((2\pi)^m \det \sum_i)^{1/2}} \exp(-\frac{1}{2} e_i^T \sum_i^{-1} e_i) \quad \text{(S4)}$$

From Equation (S4), we can obtain the likelihood function

$$L(\theta_i, \sigma_i^2) = p(\theta_i, \sigma_i^2) = \frac{1}{(2\pi\sigma_i^2)^{m/2}} \exp(-\frac{(X_i - \Phi_i\theta_i)^T (X_i - \Phi_i\theta_i)}{2\sigma_i^2}) \quad \text{(S5)}$$

Maximum likelihood estimation method aims at finding θ_i and σ_i^2 to maximize the likelihood function in Equation (S5). In order to simplify the computation, it is practical to take the logarithm of the likelihood function, which yields the following log-likelihood function:

$$\log L(\theta_i, \sigma_i^2) = -\frac{m}{2} \log(2\pi\sigma_i^2) - \frac{1}{2\sigma_i^2} \sum_{n=1}^{m} [y_i[n] - \phi_i[n] \cdot \theta_i]^2 \quad \text{(S6)}$$

where $x_i[n]$ and $\phi_i[n]$ are the *n*-th element of X_i and Φ_i in Equation (S3), respectively.

By the maximum likelihood parameter estimation method, we expect the log-likelihood function to have the maximum at $\theta_i = \hat{\theta}_i$ and $\sigma_i^2 = \hat{\sigma}_i^2$. The necessary conditions for the maximum likelihood estimates $\hat{\theta}_i$ and $\hat{\sigma}_i^2$ must conform to the following two equations.

$$\frac{\partial \log L(\theta_i, \sigma_i^2)}{\partial \theta_i} = 0$$
$$\frac{\partial \log L(\theta_i, \sigma_i^2)}{\partial \sigma_i^2} = 0 \quad \text{(S7)}$$

The estimated parameters $\hat{\theta}_i$ and $\hat{\sigma}_i^2$ are shown below,

$$\hat{\theta}_i = (\Phi_i^T \Phi_i)^{-1} \Phi_i^T Y_i \quad \text{(S8)}$$

$$\hat{\sigma}_i^2 = \frac{1}{m} \sum_{n=1}^{m} [x_i[n] - \phi_i[n] \cdot \hat{\theta}_i]^2 = \frac{1}{m} (X_i - \Phi_i \cdot \hat{\theta}_i)^T (X_i - \Phi_i \cdot \hat{\theta}_i) \quad \text{(S9)}$$

where X_i and Φ_i can be obtained from the microarray in the rough PPIN. Since there are two data sets of microarray data, two association parameters for cancer and non-cancer were separately identified.

S2. Determination of Significant Protein Associations by Akaike Information Criterion (AIC) and Student's *t*-Test

When association parameters of all the proteins in rough PPIN were identified as Equation (2), significant protein associations were determined by parameter estimates $\hat{\alpha}_{ij}$ of their association abilities. In order to determine whether the association was significant or not, Akaike Information Criterion (AIC) and Student's *t*-test, which is used to calculate the *p*-values of the association abilities, are employed to detect the system model order (or the number of model parameters) and determine the significance of our model parameters. The AIC, a method for model order detection, attempts to include both the estimated residual variance and model complexity in one statistic. AIC decreases as residual variance decreases, and increases as the number of parameters increases. As the expected residual variance decreases with increasing parameter numbers for excessive model complexity, a minimum should appear near the correct parameter number. Thus, the AIC criterion, in which estimated parameters were obtained above, was used to select model structure. Due to computation efficiency, it is impractical to compute the AIC statistics for all possible regression models. Here, the stepwise regression method which combines forward selection method and backward elimination method was applied to compute the AIC statistics. Once the estimated association parameters were examined using the AIC model detection criteria, the student's t-test was employed to calculate the *p*-values for the association abilities under the null hypothesis $H_0 : \hat{\alpha}_{ij} = 0$ to determine the significant protein associations. The *p*-values computed were then adjusted by Bonferroni correction to avoid a lot of spurious positives. The associations which adjusted *p*-value ≤0.05 were determined as significant associations and were preserved in the protein association network.

Briefly, we use the AIC method to obtain how many system orders, which mean the numbers of interactions, in the dynamic system of the association abilities (model). We use the above maximum likelihood estimate method to identify the parameter $\hat{\alpha}_{ij}$ and then employ AIC and student t-test to calculate *p*-values of association abilities for determining the significant PPIs for the target protein *i* by pruning the insignificant PPIs.

S4. MetaCore

MetaCore includes a manually annotated database of gene interactions and metabolic reactions obtained from scientific literature including the most new updating ones. The enrichment analysis of the biological process was based on the hypergeometric distribution algorithm and relevant pathway maps. Both of them are based on their statistical significance.

(i) Shortest Paths

Builds a network consisting of shortest paths between pairs of initial objects in each direction, using standard Dijkstra's shortest paths algorithm. There are "from" and "to" lists of objects; by default the whole list of seed nodes is taken for each of them. Canonical pathways are considered by this algorithm as a single step and are used for network building only if the list of objects used for network building contains both "from" and "to" of a given pathway. The Z-score, G-score and *p*-Value are three different scoring functions used to rank the small networks created by the network building algorithm named "Analyze Network". When viewing the most relevant networks list, you can sort the networks by a desired score, the actual size of the network, or the target size of the network by clicking on a column header.

(ii) Z-Score

Each subnetwork is associated with a Z-score which ranks the subnetworks according to saturation with the objects from the initial list of seed nodes. The Z-score ranks the subnetworks of the analyze network algorithm with regard to their saturation with genes from the experiment. A high Z-score means the network is highly saturated with genes from the experiment.

(iii) Z-Score Formula

$$\frac{r - n\frac{R}{N}}{\sqrt{n\left(\frac{R}{N}\right)\left(1-\frac{R}{N}\right)\left(1-\frac{n-1}{N-1}\right)}} \quad (5)$$

Legend:
N—total number of nodes in MetaCore™ database;
R—number of the network objects corresponding to the genes and proteins in your list;
n—total number of nodes in each small network generated from your list;
r—number of nodes with data in each small network generated from your list.

(iv) G-Score

The G-score modifies the Z-score based on the number of Canonical Pathways used to build the network. If a network has a high G-score, it is saturated with expressed genes (from Z-score) and it contains many Canonical Pathways. Sorting the table by this value essentially enables you to sort the table by two factors at once.

(v) p-Value

The *p*-Values throughout MetaCore™—for maps, networks and processes—are all calculated using the same basic formula for hypergeometric distribution. The *p*-Value essentially represents the probability for a particular mapping of an experiment to a map (or network, or process, *etc.*) to arise by chance, considering the numbers of genes in experiment versus the number of genes in the map (resp. network, process, *etc.*) within the "full set" of all genes on maps (resp. networks, processes, *etc.*).

p-Value Formula

$$p - Value = \frac{R!\,n!\,(N-R)!\,(N-n)!}{N!} \sum_{i=max(r,R+n-N)}^{min(n,R)} \frac{1}{i!\,(R-i)!\,(n-i)!\,(N-R-n+i)!} \quad (6)$$

Legend:
N—total number of nodes in MetaCore™ database;
R—number of the network objects corresponding to the genes and proteins in your list;
n—total number of nodes in each small network generated from your list;
r—number of nodes with data in each small network generated from your list.
